# Histone modifications and p53 binding poise the *p21* promoter for activation in human embryonic stem cells

**DOI:** 10.1038/srep28112

**Published:** 2016-06-27

**Authors:** Yoko Itahana, Jinqiu Zhang, Jonathan Göke, Leah A. Vardy, Rachel Han, Kozue Iwamoto, Engin Cukuroglu, Paul Robson, Mahmoud A. Pouladi, Alan Colman, Koji Itahana

**Affiliations:** 1 Cancer & Stem Cell Biology Program, Duke-NUS Medical School, 8 College Road, 169857 Singapore; 2 Stem Cell Disease Models A^*^STAR Institute of Medical Biology, 8A Biomedical Grove, Immunos, 138648 Singapore; 3 Stem Cell and Regenerative Biology, Genome Institute of Singapore, 60 Biopolis Street, Genome Building, 138672 Singapore; 4 Translational Laboratory in Genetic Medicine, A^*^STAR, 8A Biomedical Grove, Immunos, 138648 Singapore; 5 Computational & Systems Biology Genome Building, 138672 Singapore; 6 Translational Regulation in Stem Cells, A^*^STAR Institute of Medical Biology, 8A Biomedical Grove, Immunos, 138648 Singapore; 7 School of Biological Sciences, Nanyang Technological University, 637551 Singapore; 8 Department of Medicine, Yong Loo Lin School of Medicine, National University of Singapore, 117597 Singapore

## Abstract

The high proliferation rate of embryonic stem cells (ESCs) is thought to arise partly from very low expression of p21. However, how *p21* is suppressed in ESCs has been unclear. We found that p53 binds to the *p21* promoter in human ESCs (hESCs) as efficiently as in differentiated human mesenchymal stem cells, however it does not promote *p21* transcription in hESCs. We observed an enrichment for both the repressive histone H3K27me3 and activating histone H3K4me3 chromatin marks at the *p21* locus in hESCs, suggesting it is a suppressed, bivalent domain which overrides activation by p53. Reducing H3K27me3 methylation in hESCs rescued *p21* expression, and ectopic expression of p21 in hESCs triggered their differentiation. Further, we uncovered a subset of bivalent promoters bound by p53 in hESCs that are similarly induced upon differentiation in a p53-dependent manner, whereas p53 promotes the transcription of other target genes which do not show an enrichment of H3K27me3 in ESCs. Our studies reveal a unique epigenetic strategy used by ESCs to poise undesired p53 target genes, thus balancing the maintenance of pluripotency in the undifferentiated state with a robust response to differentiation signals, while utilizing p53 activity to maintain genomic stability and homeostasis in ESCs.

Embryonic stem cells (ESCs) are derived from the inner cell mass of blastocysts and can serve as progenitors for all adult tissues. In culture, they retain latent differentiation abilities while remaining undifferentiated, proliferative and genetically pristine. Therefore, ESCs must have extensive mechanisms for maintaining these properties. Such mechanisms could involve the tumor suppressor p53, which is expressed in ESCs. Lack of p53 has been shown to cause aneuploidy and genetic instability in ESCs^1^. In addition, p53 appears to either promote^2^ or inhibit differentiation^3^,^4^,^5^ depending on the context. p53 also serves as a barrier to the induced reprogramming of somatic cells, suggesting the pro-differentiation role of p53^6^,^7^,^8^. It remains unclear how p53 executes these two opposite functions and manages to maintain genomic stability of ESCs.

In somatic cells, p53 induces expression of *p21*, which in turn triggers cell cycle arrest in response to genotoxic stress. However, the levels of p21 in ESCs are extremely low despite the presence of p53^9^,^10^,^11^, and ESCs do not demonstrate p21-dependent or any other type of G1/S cell cycle checkpoint arrest^9^,^12^. These findings suggest that some p53 target genes are specifically repressed in ESCs.

ESCs exhibit a very high activity of cyclin dependent kinase2 (CDK2) throughout the cell cycle, and this activity is the main driving force behind the rapid proliferation of ESCs^13^,^14^,^15^,^16^. ESCs have a very short G1 phase, and lengthening the G1 phase of human ESCs leads to differentiation^10^,^17^,^18^,^19^. Therefore, suppression of p21, an inhibitor of CDK2, could allow ESCs to rapidly proliferate. In addition, since p21 has been known to inhibit apoptosis by inducing cell cycle arrest^20^,^21^,^22^, repressing p21 expression would facilitate apoptosis in ESCs suffering DNA damage, thereby preserving the genomic integrity of the cell population^9^,^23^,^24^. However, how p21 expression is controlled in ESCs is currently unclear.

p53 is a cellular stress sensor that triggers cell cycle arrest, apoptosis and senescence dependent on cellular context. Recent evidence highlights the tumor suppressive roles of p53 in modulating diverse downstream pathways, such as cellular metabolism, antioxidant and autophagy in unstressed cells^25^. However, little is known about the role of these diverse p53 functions in maintaining the quality of ESCs.

Here, we have identified a mechanism that suppresses p21 in hESCs. Although p53 localizes to the nucleus and binds to the *p21* promoter in hESCs as efficiently as in differentiated mesenchymal stem cells, *p21* transcription is suppressed by histone H3K27 trimethylation specifically in hESCs. Depletion of this modification in hESCs by the pharmacological inhibitor DZNep induces p21 expression, and ectopic expression of p21 induces differentiation of hESCs. Interestingly, p53 promotes the transcription of a diverse subset of target genes which do not show an enrichment of H3K27me3 in hESCs, whereas another subset, including *p21*, is enriched for bivalent chromatin marks and poised for p53-dependent activation upon differentiation. Our study reveals a unique epigenetic strategy in ESCs to selectively regulate different p53 target genes and prepare for robust differentiation signals.

## Results

### p21 regulation by p53 is attenuated in hESCs and hiPSCs

**Figure 1:**
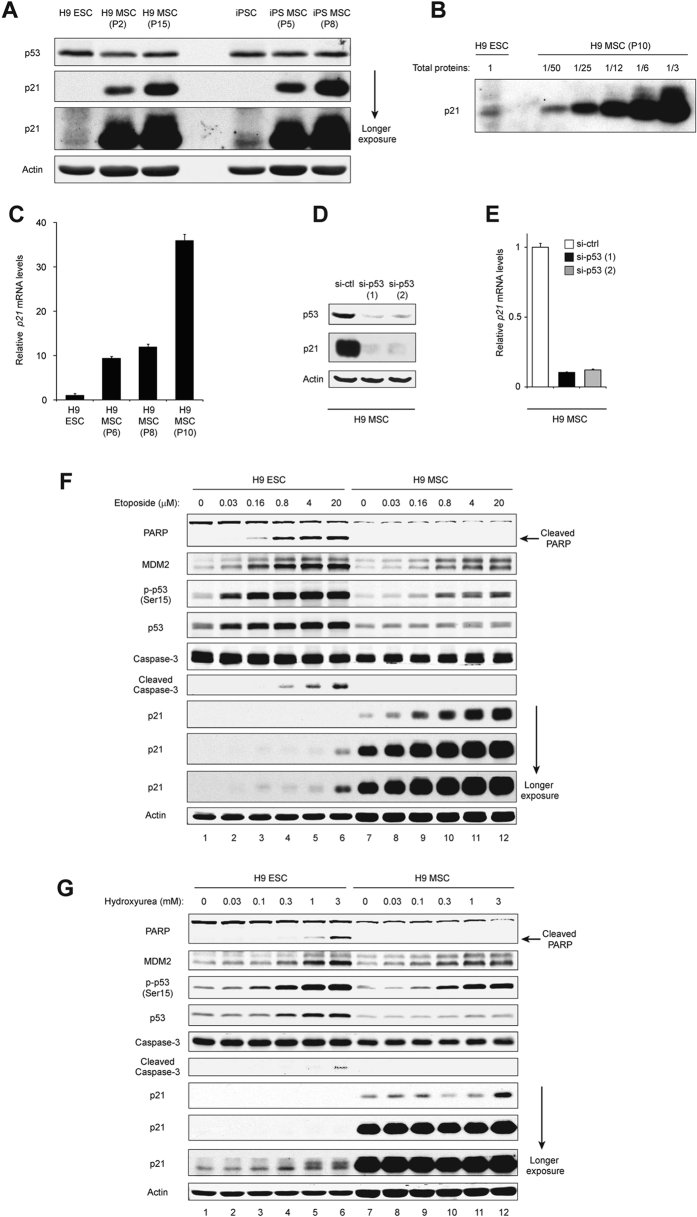
p21 expression is suppressed in human embryonic stem cells. (**A**) p21 expression is suppressed in hESCs and hiPSCs compared to hMSCs. Protein lysates from the indicated cells were analyzed by Western blotting with the indicated antibodies. The passage number is shown in brackets. 70 μg of protein lysate was loaded in each lane. (**B**) p21 expression in hESCs is about 50 times lower than in hMSCs, as analyzed by Western blotting with the indicated antibody. 150 μg of protein lysate from H9 hESCs was loaded in lane 1. The amount of total protein lysate loaded relative to hESC is indicated. (**C**) *p21* mRNA levels are lower in H9 hESCs than in hMSCs, as assessed by qRT-PCR (n = 3, means ± SD). The mean value of mRNA expression in H9 hESCs is set at 1, and relative expression is shown. *TBP* was used as an internal control for normalization. (**D,E**) p53 is required for p21 expression in H9 hMSCs (passage number 8). H9 hMSCs were transfected with control and p53 siRNAs. p21 protein levels (**D**) were analyzed by Western blotting. 50 μg of protein lysate was loaded in each lane. *p21* mRNA levels (**E**) were analyzed as in (**C**). The mean value of mRNA expression in control siRNA transfected cells is set at 1, and relative expression is shown. (**F,G**) p21 expression in H9 hESCs remains very low upon p53 activation by DNA damage. H9 hESCs and H9 hMSCs were treated with the indicated concentration of etoposide (**F**) or hydroxyurea (**G**) for 24 hrs and harvested for Western blotting. The passage numbers of H9 hESCs and hMSCs are P37 and P10 respectively. 50 μg of protein lysate was loaded in each lane.

Since p53 is a well-established regulator of p21 expression, we compared the expression of p53 and p21 in human ESCs (hESCs), induced pluripotent cells (hiPSCs) and mesenchymal stem cells (hMSCs). N1-hiPSCs were generated from normal adult fibroblasts by reprogramming using the four Yamanaka factors, whereas hMSCs were generated by differentiating H9 hESCs or N1-hiPSCs respectively^26^. H9 hESCs and H9 hMSCs were validated by using several pluripotent and differentiation markers respectively (Fig. S1A,B). The validation of N1-hiPSCs and N1-hiPS MSCs were previously described^26^. Although p53 protein levels were similar between hESCs and hMSCs, p21 levels were barely detectable in hESCs and high in hMSCs, consistent with earlier reports^9^,^12^ (Fig. 1A). p21 levels increased even further in later passages of hMSCs (Fig. 1A, lane 2 and lane 3). Similar results were obtained with hiPSCs (Fig. 1A). By comparing signals from serially diluted protein lysates, we estimated that hMSCs express up to 50 times more p21 than H9 hESCs depending on the passage numbers (Fig. 1B). *p21* mRNA levels were also substantially higher in hMSCs relative to hESCs (Fig. 1C), consistent with the difference in p21 protein expression between these cells. To determine if p21 expression in hMSCs requires p53, we used RNAi to repress p53. Knockdown of p53 in hMSCs drastically reduced p21 protein and mRNA levels (Fig. 1D,E). These results suggest that p53 significantly contributes to the expression of p21 in hMSCs, but the similar levels of p53 protein expression are not sufficient to induce the same level of p21 expression in hESCs.

We next asked if p21 expression would reach the levels observed in hMSCs upon activation of p53 in hESCs. To activate p53, we induced DNA damage by treating cells with increasing concentrations of etoposide, a topoisomerase inhibitor. Etoposide triggered Ser15 phosphorylation of p53 in both H9 hESCs and H9 hMSCs (Fig. 1F), indicating that the stress response pathway upstream of p53 is intact in both cells. ESCs are highly sensitive to DNA damage and undergo apoptosis. In fact, increasing concentrations of etoposide induced PARP cleavage and caspase-3 cleavage in H9 hESCs (0.16 μM to 20 μM, lane 3 to 6), but not in H9 hMSCs (lane 9 to 12). To compare p21 expression in hESCs and hMSCs without apoptosis, we examined H9 hESCs with the lowest dose of etoposide (0.03 μM) (Fig. 1F, lane 2). p53 Ser15 phosphorylation levels were comparable between H9 hESCs treated with 0.03 μM etoposide (lane 2) and H9 hMSCs with 20 μM etoposide (lane 12). Importantly, when we compared these two conditions (lanes 2 and 12), p21 was markedly induced only in H9 hMSCs (lane 12), and the expression of p21 in H9 hESCs remained very low with 0.03 μM etoposide (lane 2). Interestingly, MDM2, another well-known p53 target gene product, expressed similarly in H9 hESCs and hMSCs, and increasing concentrations of etoposide induced MDM2 comparably in H9 hESCs and H9 hMSCs, suggesting that the expression of p21, but not MDM2, is selectively suppressed in H9 hESCs, even if p53 is activated.

We next used hydroxyurea as an alternative DNA damage reagent to activate p53 and confirmed our findings with etoposide. Hydroxyurea depletes dNTPs from cells, resulting in stalled replication forks^27^ which eventually collapse and generate DNA double strand breaks after prolonged treatment^27^. Hydroxyurea (HU) is known to activate p53 and induce p53 target genes^28^. Similar to etoposide treatment, HU induced p21 expression in hMSCs (Fig. 1G, lane 12), whereas the expression of p21 was still very low in H9 hESCs (Fig. 1G, lane 1–6) even if Ser15 phosphorylation was efficiently induced (lane 4–6). On the other hand, MDM2 was induced by HU similarly in H9 hESCs and hMSCs. Similar results were obtained using N1-hiPSCs and N1-hiPS MSCs after treatment with etoposide (Fig. S2A) or HU (Fig. S2B). Other post-translational modifications of p53, such as methylation at K372 and acetylation at K120 and K382, were also comparable between H9 hESCs and hMSCs (Fig. S3). These data suggest that, although the pathway activating p53 is intact, the expression of p21 is selectively suppressed in hESCs and hiPSCs compared to hMSCs, regardless of the presence or absence of stress.

### Protein stability and translational efficiency do not contribute substantially to the low expression of p21 in hESCs

**Figure 2:**
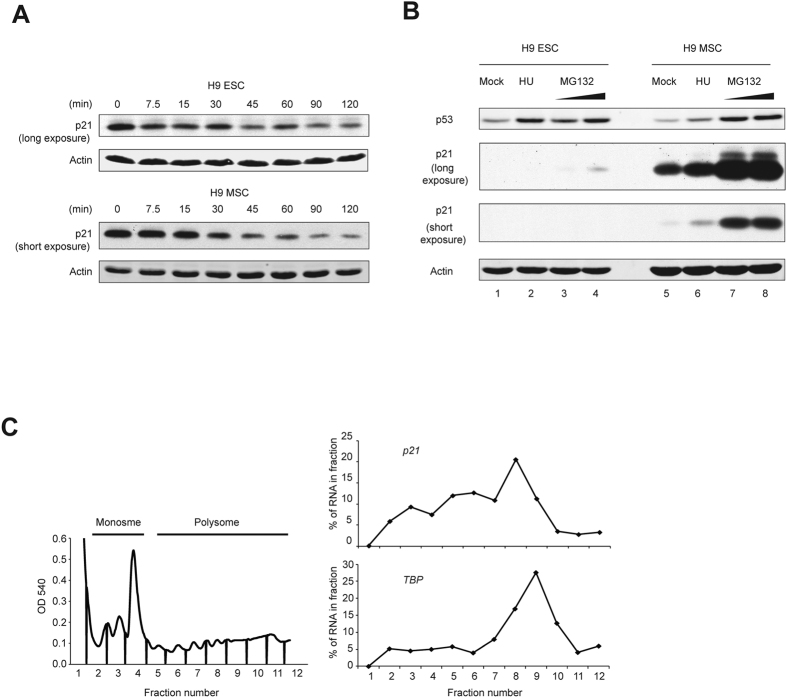
Protein stability and translational efficiency do not underlie low levels of p21 protein in hESCs. (**A**) The half-life of the p21 protein is comparable in H9 hESCs and hMSCs. Cells were treated with cycloheximide and cell lysates were harvested at the indicated time for Western blotting analysis. An increased amount (250 μg) of lysate and longer exposure time were used to enable detection of p21 in hESCs. 50 μg of protein lysate was used for hMSCs. The passage numbers of H9 hESCs and hMSCs are P46 and P5 respectively. (**B**) The p21 protein is not subject to increased proteasomal degradation in hESCs relative to hMSCs. Cells were treated with MG132 or hydroxyurea, and cell lysates were analyzed by Western blotting. The passage number of H9 hESCs and hMSCs are P46 and P6 respectively. 50 μg of protein lysate was loaded in each lane. (**C**) Translation state analysis of *p21* mRNA in H9 hESCs. Representative polysome profile from H9 hESCs indicating monosomes and polysome fractions is shown in the left panel. qRT-PCR analysis shows the percentage of *TBP* and *p21* mRNA in each fraction in the right panels.

To investigate additional mechanisms that may underlie the relatively low p21 levels in hESCs, we assessed protein stability and translational efficiency. We treated cells with cyclohexamide to inhibit protein synthesis and found that the half-life of the p21 protein was similar in hESCs and hMSCs (Fig. 2A). We also treated both hESCs and hMSCs with the proteasome inhibitor MG132, and monitored the levels of p21. Although p21 protein levels increased slightly upon treating H9 hESCs with MG132 (Fig. 2B, lane 1 and 4), they were still much lower than in H9 hMSCs, with or without MG132 treatment (Fig. 2B, lane 4, 5, 7, and 8). These results suggest that enhanced proteasomal degradation does not account for the low levels of p21 in hESCs. Further, we used polysome profile analysis to assess the translational efficiency of *p21*. *p21* mRNA was enriched in the polysome fractions, suggesting that it is being actively translated in hESCs (Fig. 2C, right panel). *p21* mRNA was similarly enriched on the polysomes in WI-38 cells (Fig. S4A), a normal human fibroblast cell line which has similar p21 mRNA and protein levels as H9 hMSCs (Fig. S4B). *TBP*, a house keeping gene, was enriched in the polysomes to a similar extent as *p21* in both cell lines (Figs 2C and S4A). Taken together, these data suggest that post-translational and translational mechanisms do not contribute substantially to the low expression of p21 in hESCs.

### *p21* is transcriptionally silent but poised for p53-dependent activation in hESCs

**Figure 3:**
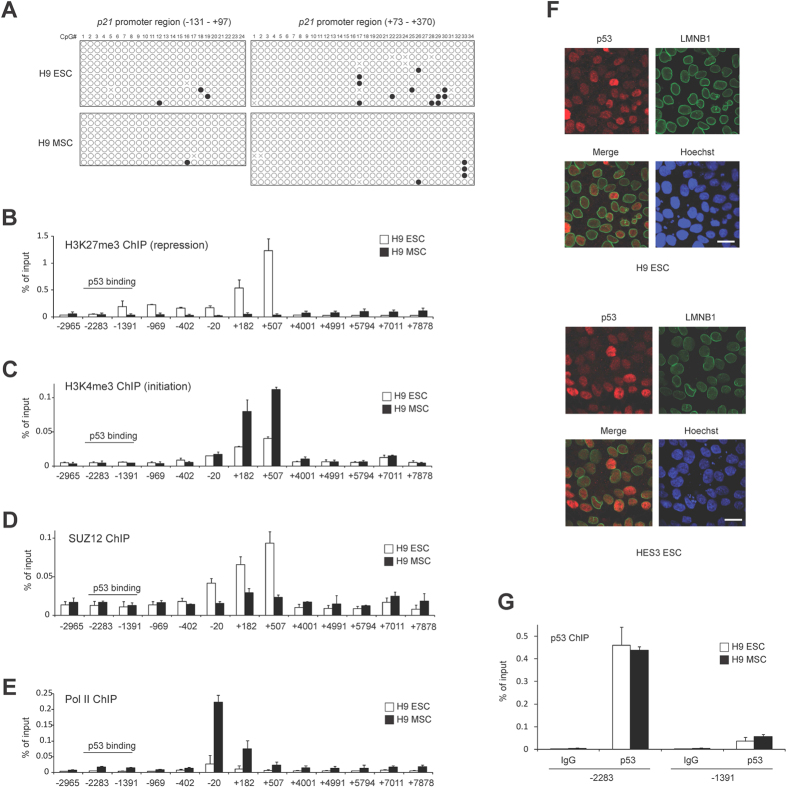
p21 expression in hESCs is repressed by histone H3K27me3. (**A**) *p21* promoter DNA is not significantly methylated in H9 hESCs and hMSCs. The CpG rich promoter regions of *p21* (−131− + 97 bp and +73 − + 370 bp in relation to the transcriptional start site, TSS) were subjected to bisulfite sequencing. Each line shows independent sequencing results (n = 8–11). Filled circles indicate methylated CpGs and open circles represent unmethylated CpGs (X denotes un-identified nucleotide sequence). Clones with at least 90% cytosine conversion were considered as unmethylated sites. (**B–E**) ChIP analysis on the *p21* gene locus was performed using protein lysates of H9 hESC and hMSCs with the indicated antibodies. ChIP-enriched DNA was quantified by qPCR (n = 3, mean ± SD). Values are shown as a percentage of input DNA. The amplicon’s name indicates the position of the central base pair of the amplicon relative to the TSS of *p21* gene. Note that *p21* gene locus is silenced by H3K27me3. (**F**) p53 localizes in the nucleus. Immunofluorescence staining of p53 and LMNB1 (nuclear envelope marker) in hESC line H9 and HES3. Scale bar, 20 μm. (**G**) p53 protein binds equally well to the *p21* promoter in H9 hESC and hMSC. ChIP analysis with p53 antibody was performed as in (**B**). Mouse IgG was used as the negative control for immunoprecipitation.

Given that *p21* mRNA levels are lower in hESCs than in hMSCs, we hypothesized that *p21* transcription is repressed in hESCs. To determine if the *p21* promoter is silenced by DNA methylation in hESCs, we performed bisulfite sequencing. Bisulfite treatment of both hESCs and hMSCs converted most of the cytosines to uracil in the *p21* promoter region between −131 and +370 bp in relation to the transcriptional start site, indicating a lack of cytosine methylation (Fig. 3A). Thus, *p21* is not suppressed by DNA methylation in hESCs.

Recent genome-wide chromatin studies have revealed that a subset of genes in hESCs have “bivalent promoters”, which are modified by both the activating histone H3K4 trimethyl (H3K4me3) and the repressive histone H3K27 trimethyl (H3K27me3) marks^29^. Most bivalent genes are required for development, and are poised for future expression depending on different developmental signals. To determine if histone modifications might regulate *p21* expression, we performed ChIP to evaluate H3K27me3 and H3K4me3 at the *p21* locus in hESCs and hMSCs. H3K27me3 was enriched around the transcriptional start site (TSS) in hESCs but not hMSCs (Fig. 3B), whereas H3K4me3 was evident in both hESCs and hMSCs (Fig. 3C). Since trimethylation of H3K27 has been shown to be mediated by the Polycomb Repressive Complex 2 (PRC2), we performed ChIP for the PRC2 component SUZ12. We observed SUZ12 enrichment at the +182 and +507 bp regions of *p21* in hESCs but not hMSCs, which overlap with the regions of H3K27me3 enrichment (Fig. 3D). To further evaluate the transcriptional activity at the *p21* locus, we performed ChIP for RNA polymerase II (Pol II). We found that Pol II was depleted around TSS in hESCs compared to hMSCs (Fig. 3E). Together, these data suggest that *p21* has a transcriptionally silent and bivalent promoter in hESCs that is poised for activation.

To determine if p53 associates with the *p21* promoter in hESCs, we examined its subcellular and genomic localization. Some previous reports have claimed that p53 is localized exclusively in the cytoplasm in mouse ESCs and suggested that this impedes its role as a transcription factor^3^,^11^,^30^. However, other recent reports using human and mouse ESCs showed that p53 is mainly nuclear^24^,^31^,^32^. We evaluated the subcellular distribution of endogenous p53 in the human ESC lines H9 and HES3 and mouse ESC line E14, and found nuclear localization in both human ESCs (Fig. 3F) and mouse ESCs (Fig. S5A). In addition, exogenous p53, introduced by lentivirus infection into H9 hESCs, was also detected in the nucleus (Fig. S5B). Next, we examined the occupancy of p53 at the *p21* promoter in hESCs and hMSCs. The *p21* promoter possesses two p53 binding sites, around −2283 bp (high affinity binding site) and −1391 bp (low affinity binding site)^33^,^34^. Unexpectedly, p53 bound equally well to the *p21* promoter in both cell lines (Fig. 3G), indicating that p53 associates with the *p21* promoter in hESCs but cannot activate transcription efficiently. Thus, although the *p21* promoter is epigenetically silenced in hESCs, both the bivalent chromatin state and binding of p53 poise it for activation upon differentiation.

### Reducing H3K27me3 levels restores p21 expression in hESCs

**Figure 4:**
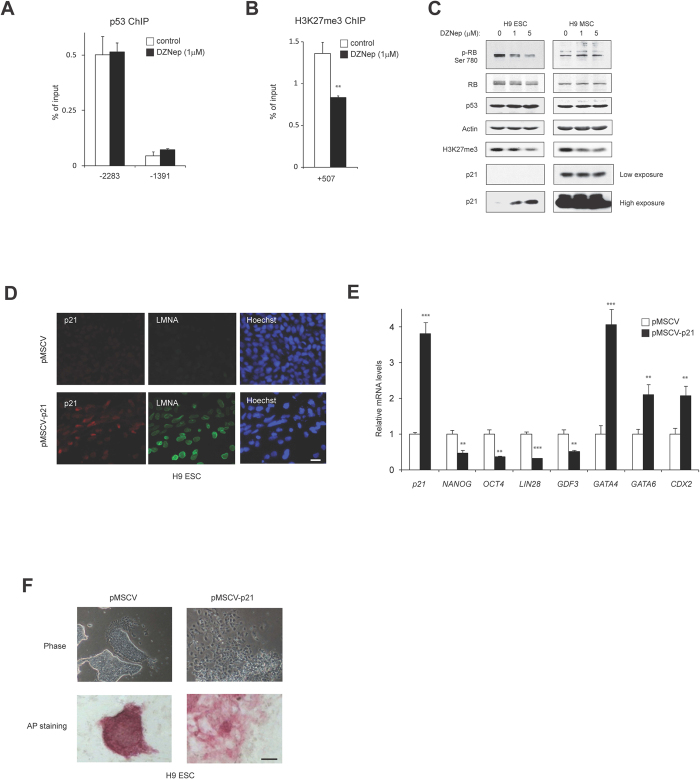
DZNep rescues p21 expression in hESCs, and p21 expression in hESCs induces differentiation. (**A,B**) DZNep reduces H3K27me3 marks on the *p21* locus without affecting p53 binding to the *p21* promoter. H9 hESCs were treated with DZNep (1 μM) for 2 days and ChIP analysis was performed using p53 antibody (**A**) or H3K27me3 antibody (**B**) as in Fig. 3 (n = 3, mean ± SD). **p < 0.01, two-tailed unpaired *t*-test compared to control cells. The passage number of H9 hESCs is P35. (**C**) DZNep induces the expression of p21 in hESCs. H9 hESCs and hMECs were treated with DZNep at the indicated dose for 2 days. Proteins were harvested for Western blotting analysis with the indicated antibodies. The passage numbers of H9 hESCs and hMSCs are P35 and P9 respectively. (**D**) Ectopic p21 expression in hESCs. H9 hESCs were infected with retrovirus carrying either empty (pMSCV) or p21 and selected with blasticidin for 3 days. Immunofluorescence staining of p21 and differentiation marker, LMNA are shown. Scale bar, 20 μm. (**E**) Introduction of p21 reduces the expression of pluripotency markers and increases the differentiation markers in H9 hESCs. The mRNA expression of the indicated genes was analyzed as in Fig. 1C. The mean value of mRNA expression in pMSCV infected cells is set at 1, and relative expression is shown. **p < 0.01, ***p < 0.001, two-tailed unpaired *t*-test compared to control cells. (**F**) Expression of p21 in hESCs triggers a differentiated cell morphology (Phase) and reduces alkaline phosphatase staining (AP staining). Scale bar, 100 μm.

To determine if H3K27me3 is required to repress the *p21* promoter in hESCs, we treated cells with 3-Deazaneplanocin A (DZNep), which was previously shown to reduce the levels of PRC2 components, such as EZH2 and SUZ12, and to selectively block H3K27 methylation^35^,^36^. As expected, DZNep treatment reduced the levels of EZH2 and SUZ12 in both hESCs and hMSCs (Fig. S6). DZNep did not affect the binding of p53 to the *p21* promoter (Fig. 4A), however it reduced H3K27me3 enrichment at the *p21* gene at +507 bp (Fig. 4B) and it induced p21 expression in hESCs (Fig. 4C). In contrast, although DZNep treatment similarly decreased total levels of H3K27me3 in hESCs and hMSCs, it had no effect on p21 expression in hMSCs (Fig. 4C), consistent with the lack of H3K27me3 at the *p21* gene in hMSCs (Fig. 3B). p21 inhibits CDK2 and CDK4/6, which phosphorylate the retinoblastoma protein (RB) for S-phase entry. Interestingly, DZNep treatment decreased the levels of phosphorylated RB in hESCs (Fig. 4C), consistent with the increased expression of p21. Thus, reducing H3K27 trimethylation by DZNep activates p21 expression in hESCs.

### p21 induces differentiation of hESCs

Our results reveal that *p21* expression is suppressed by epigenetic silencing in hESCs. To determine how ectopic p21 expression would affect hESCs, we introduced exogenous p21 by retroviral infection of H9 hESCs. p21 expression was verified by immunofluorescence and qRT-PCR (Fig. 4D,E). We achieved moderate ectopic *p21* expression levels that were about 4 times higher than endogenous levels but less than that seen in hMSCs (Figs 1C and 4E). Ectopic expression of p21 significantly reduced the levels of pluripotency markers (*NANOG, OCT4, LIN28* and *GDF3*) and increased the levels of differentiation markers (*GATA4, GATA6* and *CDX2*) in H9 hESCs (Fig. 4E). Another differentiation marker, Lamin A/C (LMNA), also increased the expression by p21 introduction (Fig. 4D). In addition, H9 hESCs expressing ectopic p21 displayed a more differentiated cell morphology compared to the control cells, consistent with the reduced alkaline phosphatase staining of these cells (Fig. 4F). Taken together, these data suggest that p21 suppression in hESCs is required to maintain their undifferentiated state.

### A subset of p53 target genes undergo epigenetic silencing in hESCs

**Figure 5:**
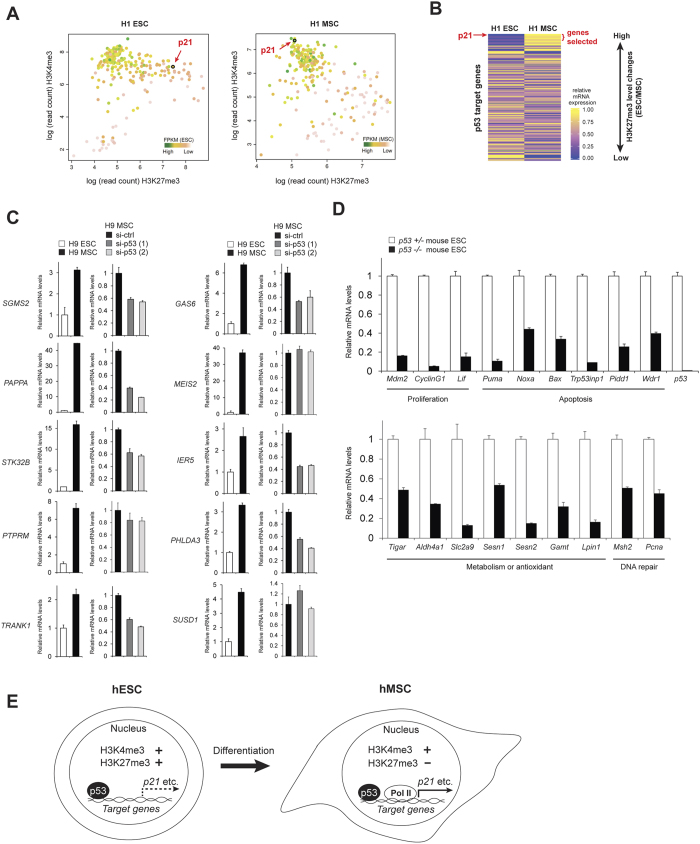
A subset of p53 target genes is subject to epigenetic silencing in hESCs. (**A**) H3K4me3 and H3K27me3 levels at the TSSs of putative p53 target genes in H1 hESCs were plotted (left). The same set of genes were also plotted for H3K4me3 and H3K27me3 levels in H1-derived hMSCs (right). The *p21* gene is marked in black open circle and shows high levels of H3K27me3 in hESCs, indicating that it is epigenetically repressed. The color corresponds to the mRNA expression level (FPKM) for each gene in the respective cell type. (**B**) Heatmap showing relative mRNA expression of p53 target gene candidates in H1 hESCs and hMSCs. Genes were sorted by the ratio of H3K27me3 levels in hESCs:hMSCs. FPKM values were normalized per gene to obtain relative expression values. The *p21* gene and the genes selected for further analysis in Fig. 5C are indicated in red. (**C**) A subset of p53 target gene candidates is suppressed in H9 hESCs and requires p53 for expression in hMSCs. RNA was extracted from H9 hESCs and hMSCs and the indicated genes were analyzed by qRT-PCR (n = 3, means ± SD) (left plot). The mean value of mRNA expression in H9 hESCs is set at 1, and relative expression is shown in the left plot. H9 hMSCs were transfected with control and p53 siRNAs, and mRNA levels of each gene were analyzed similarly (right plot). The mean value of mRNA expression in control siRNA transfected H9 MSCs is set at 1, and relative expression is shown in the right plot. The passage number of H9 hMSCs is P8. (**D**) A subset of known p53 target genes is expressed in a p53-dependent manner in mouse ESCs. RNA was extracted from mouse p53^+/−^ ESCs and littermate p53^−/−^ ESCs, and the indicated genes was analyzed as in (**C**). The mean value of mRNA expression in p53^+/−^ ESCs is set at 1, and relative expression is shown. (**E**) A conclusion model: A subset of p53 target genes such as *p21* is poised but silenced by bivalent promoters in hESCs, and activated by releasing H3K27me3 marks upon differentiation.

We next examined if there are other p53 target genes that may be similar to *p21* and are epigenetically silenced in hESCs. We used existing ChIP-Seq data sets of p53^37^ from hESCs and H3K27me3^38^ and H3K4me3^38^ from hESCs and hMSCs to analyze H3K27me3 and H3K4me3 enrichment at transcription start sites (TSS) with nearby p53 binding (within 50 kb). Interestingly, only a minority of p53 target gene candidates showed H3K27me3 enrichment at the TSS in hESCs (Fig. 5A). Approximately 9% of p53 target gene candidates show higher than 2.5-fold H3K27me3 marks in hESCs compared to hMSCs, and have bivalent promoters (positive for both H3K27me3 and H3K4me3) (Fig. 5A, Supplementary Tables 1 and 2). Importantly, 95% of the p53 target gene candidates enriched for H3K27me3 in hESCs show reduced mRNA levels in hESCs compared to hMSCs by RNA-seq^38^ (Fig. 5A,B, Supplementary Table 2), suggesting that the expression of these genes is triggered by loss of H3K27me3 upon differentiation. Among them, the *p21* TSS showed one of the largest reductions in H3K27me3 levels after differentiation (Fig. 5A,B, Supplementary Table 2).

Next, we investigated if p53 is required to induce these poised p53 target gene candidates upon differentiation, as it is for *p21*. We selected the top 10 p53 target gene candidates (excluding *p21*) showing the largest reductions in H3K27me3 levels after differentiation (Fig. 5B and Supplementary Table 2). We verified that all 10 genes displayed higher expression in hMSCs compared to hESCs by qRT-PCR (Fig. 5C), suggesting that these genes are silenced in hESCs by H3K27me3, despite p53 binding near their promoters. Knockdown of p53 in hMSCs reduced the expression of 7 out of 10 genes (*SGMS2, PAPPA, STK32B, TRANK1, GAS6, IER5* and *PHLDA3*; *p* < 0.01, two-tailed unpaired *t*-test) (Fig. 5C), suggesting that these 7 genes are induced in a p53-dependent manner upon differentiation, similar to *p21*.

There is also a subset of p53 target genes which were marked by H3K4me3 without an enrichment of H3K27me3 marks in hESCs, suggesting that these genes may be expressed in hESCs in a p53-dependent manner (Fig. 5A). To confirm that p53 promotes the expression of some downstream target genes for maintaining the quality of ESCs, we compared *p53*^+/−^ and littermate *p53*^*−/−*^ mouse ESCs for the expression levels of known p53 target genes that did not show a strong enrichment of H3K27me3 in hESCs^38^. The p53 target genes which are involved in the regulation of proliferation (3 genes including *Mdm2*), apoptosis (6 genes), metabolism (7 genes), and DNA repair (2 genes)^25^, were significantly reduced in *p53*^*−/−*^ ESCs compared to *p53*^+/−^ ESCs (Fig. 5D), suggesting that steady state levels of p53 actively induce these genes in ESCs.

Taken together, our findings indicate that p53 actively induces a subset of its target genes in hESCs. However, epigenetic repression regulates a subset of p53 target genes, including *p21*, to both prevent differentiation of hESCs and poise for robust expression in response to differentiation signals.

## Discussion

The repression of p21 is thought to be beneficial for ESCs to maintain rapid proliferation and genomic integrity by activating CDKs and increasing apoptosis sensitivity, respectively. However, the mechanism of p21 repression has been unclear. We found that *p21* is epigenetically silenced through H3K27me3 in hESCs. DZNep, a pharmacological inhibitor of PRC2, reduced H3K27me3 levels and induced p21 expression in hESCs. p21 expression in DZNep-treated, undifferentiated hESCs was still lower than in differentiated hMSCs, perhaps due to incomplete removal of H3K27me3 (Fig. 4B) or to the presence of other factors that contribute to *p21* repression, such as miR-302^39^. In addition, other histone modifications, such as acetylation, may also regulate *p21* expression. Nevertheless, our data suggest that *p21* belongs to a small subset of target genes bound by p53 that are epigenetically silenced by histone modifications in hESCs to support rapid proliferation, maintain genomic integrity and prevent inappropriate differentiation.

Interestingly, we found that a subset of p53 target gene candidates, including *p21*, contain the bivalent chromatin modifications H3K4me3 (activating) and H3K27me3 (repressive) in hESCs. It has been shown that there are many of these bivalent promoters in ESCs^29^. The bivalent mark is thought to confer timely activation in response to differentiation signals, suggesting that a subset of p53 target gene candidates, including *p21*, may require prompt activation upon differentiation. In fact, our results showed that the expression levels of these genes were increased in a p53-dependent manner after differentiation from hESCs to hMSCs. Further, p53 binds to the *p21* promoter equally well in hESCs and hMSCs, suggesting that p53 is equally functional as a transcription factor in these cells and that the *p21* promoter is poised for p53-dependent activation upon differentiation of hESCs (Fig. 5E). Besides *p21*, there are also other p53 target gene candidates which are poised in hESCs and upregulated upon the differentiation (Fig. 5C). For example, *PHLDA3*, Pleckstrin Homology-Like Domain, Family A, Member 3, is a well-known p53 target gene that suppresses AKT signaling^40^. AKT signaling is known to contribute to pluripotency of ESCs^41^, suggesting that *PHLDA3* may be suppressed in order to activate AKT signaling for keeping pluripotency in ESCs. SGMS2, Sphingomyelin Synthase 2, is a bidirectional sphingomyelin synthase involved in ceramide metabolism. Since ceramide is widely-used to differentiate ESCs^42^, SGMS2 may need to be suppressed to prevent unwanted differentiation in ESCs. GAS6, Growth Arrest-Specific 6, is a biological ligand of AXL receptor tyrosine kinase^43^ that is involved in the differentiation of several tissues. It has been recently showed that inhibition of AXL contributes to the maintenance of pluripotency of hESC and to the improvement of hiPSCs generation from fibroblasts^44^. Therefore, repression of GAS6 may be important for hESCs to keep their undifferentiated state.

We showed that ectopic p21 expression induces differentiation of hESCs and reduces the expression of pluripotency markers. These data are consistent with the finding that depletion of p21 enhances iPSC generation from somatic cells^6^ and suggest that p21 is an inhibitor of the pluripotent state. Consistent with our data, several reports suggested the importance of having low levels of p21 or having high activity of CDK2 in human ESCs to keep pluripotency and self-renewal. For example, ectopic expression of p21 induces hESCs differentiation^10^. CDK2 inhibition by drugs or RNA interference induce differentiation in hESCs^17^,^19^. CDK2 inhibition by p27 induces hESCs differentiation^18^. In contrast to human ESCs, the effects of p21 expression and CDK2 inhibition on mouse ESCs (mESCs) were still controversial. p21 does not induce differentiation in mESCs^45^. Consistent with this, CDK2 inhibitors do not have effect on differentiation in mESCs^13^ and knockout mice of *Cdk2* were viable although *Cdk2* is required for germ cell development^46^,^47^. On the other hand, other report showed that CDK2 inhibitor induces differentiation in mESCs^14^. Recent reports showed that p21 can facilitate the expression of differentiation marker at low serum condition in mESCs^48^, suggesting that p21 is able to tip the balance toward pro-differentiation state depends on the culture condition even in mESCs. These differences in sensitivity to CDK2 inhibition between human and mouse ESCs may come from the difference in cell cycle regulation. In fact, unlike mESCs, most of the cell cycle regulators including cyclin D3, E and A in hESCs show cell cycle phase-specific expression^19^. Nevertheless, all these reports showed that p21 expression or CDK2 inhibition in both hESCs and mESCs prolongs G1 phase and reduces rapid proliferation rate. Therefore, it is well established that the low levels of p21 is critical for short G1 and rapid proliferation that is an important characteristic of ESCs, which emphasizes the importance of having the mechanism to suppress p21 expression in ESCs.

Our results and several recent reports suggest that p53 is functional in ESCs^24^,^49^,^50^. For examples, similarly to p53-null somatic cells, chromosomal aneuploidy was observed in p53-null hESCs, suggesting that p53 plays an important role in preventing genomic instability in ESCs^1^. The canonical functions of p53 in somatic cells are the induction of cell cycle arrest, senescence and apoptosis upon cellular stress. Recent evidences challenge this long held view of p53-mediated tumor suppression and highlight the importance of non-canonical diverse functions of p53 in the absence of cellular stresses^25^,^51^,^52^. In fact, our results showed that p53 target genes involved in the regulation of proliferation, metabolism and DNA repair, are actively transcribed in a p53-dependent manner in ESCs without stress. It is highly possible that these diverse functions of p53 are also involved in the maintenance of the genomic stability and homeostasis of ESCs, which remains to be explored.

The role of p53 in differentiation and development is still unclear. Even though the majority of *p53*-null mice can be born without any noticeable developmental defects, the frequency of developmental abnormalities in *p53*-null mice is higher than wild-type mice^53^. Several reports have revealed that p53 can either suppress or promote the differentiation of ESCs, depending on the context. p53 activates Wnt signaling, which maintains ESCs in an undifferentiated state^2^. On the other hand, p53 induces differentiation by suppressing Nanog^4^, and hyperactivation of p53 by Nutlin, an inhibitor of p53-MDM2 binding, also induces differentiation^5^. It has been unclear how p53 can differentially exert both anti- and pro-differentiation functions. However, at least our data indicated that the upregulation of p21 by p53 is undesired for ESCs to maintain undifferentiated state, suggesting the existence of another layer of mechanisms to suppress the unwanted p21 expression in ESCs. Based on our data, we propose a model in which, while p53 actively induces genes involved in the maintenance of genomic stability and homeostasis of ESCs, a subset of p53 target genes, like *p21*, are bound by p53 but regulated by epigenetic marks that prevent p53-mediated activation in ESCs (Fig. 5E). These genes may otherwise induce undesired changes in ESCs, such as differentiation, preventing apoptosis downstream of DNA damage, decreasing proliferation rate and changing cell cycle profiles. In response to proper signal of differentiation, H3K27me3 marks are removed and p53 rapidly induces these genes (Fig. 5E). Elucidating how these genes are targeted for specific histone modifications and the consequences of removing their epigenetic marks will further our understanding of pluripotency, metabolism and genomic integrity in ESCs.

## Materials and Methods

### Cell Lines and Cell Culture

Human ESCs, H9 and HES3 were purchased from WiCell. H9 hESCs were verified by immunofluorescence staining with pluripotent markers OCT4, NANOG, SOX2, SSEA-4, and TRA-1-80, and by flow cytometry analysis of SSEA-4 and TRA-1-80 using PE- or FITC-conjugated antibodies (BD Biosciences 1:50) (Fig. S1A). N1-hiPSCs were derived from normal adult fibroblasts by retroviral infection of the four Yamanaka factors (OCT4, SOX2, KLF4 and MYC) and were verified by immunofluorescence staining with pluripotent markers OCT4, NANOG, SOX2, SSEA-4, and TRA-1-80, and by teratoma formation as shown in our previous publication^26^. Mouse ESCs E14 was purchased from ATCC (CRL-1821). *p53*^+/−^ (p2.2) and littermate *p53*^*−/−*^ (p1.1) mouse ESCs were kindly provided by Dr. Kanaga Sabapathy (National Cancer Centre Singapore, Singapore) and generating these cell lines has been described previously^23^. hESCs and hiPSCs were cultured on matrigel (BD Biosciences) with mTesR1 medium (Stem Cell Technologies). mESCs were maintained with 20% FBS in DMEM medium with nonessential amino acid, 2-mercaptoethanol, penicillin/streptomycin, glutamine and LIF. All cells were routinely maintained in a 37 °C incubator with 5% CO_2_. H9 hESCs with passage number between 29 and 52 were used in the experiments unless otherwise indicated. N1-hiPSCs with passage number between 36 and 39 were used in the experiments unless otherwise indicated.

### Differentiation of hESCs and hiPSCs into hMSCs

H9 hESCs and N1 hiPSCs were differentiated into hMSCs according to the protocol previously described^54^. hMSCs were purified by cell sorting of CD105 + /CD24- and maintained with medium containing 90% DMEM, 10% fetal calf serum, and 5 ng/ml bFGF (Life Technologies, Gibco). Identity of H9 hMSCs was verified by flow cytometry analysis of MSC surface markers CD29, CD44, CD73 and CD105 using PE- or FITC-conjugated antibodies (all BD Biosciences 1:50) (Fig. S1B). Identity of N1-hiPS-MSCs was verified by flow cytometry analysis of MSC surface markers CD29, CD44, CD73, CD105 and CD166 as shown in our previous publication (BD Biosciences)^26^. BD FACSCalibur was used for detection and quantitative analysis. H9 hMSCs with passage number between 2 and 16 were used in the experiments unless otherwise indicated.

### Reagents

Hydroxyurea, MG132, cycloheximide, etoposide and heparin were purchased from Sigma. DZNep was kindly provided by Dr. Qiang Yu (Genome Institute of Singapore, Singapore). MG132 was used at 1 μM and 2 μM for hESCs and at 2 μM and 20 μM for hMSCs for 5 hrs. Hydroxyurea was used at concentrations between 0.03 mM and 3 mM in Figs. 1G and S2B and at 20 mM in Fig. 2B.

### Retrovirus Production and p21 overexpression

The pMSCV-based retroviral vector encoding human *p21* was obtained from Dr. Mathijs Voorhoeve (Duke-NUS Medical School, Singapore). Retrovirus was produced as described^55^. H9 hESCs were transduced with empty virus and p21 virus respectively for 7–12 hours in 3 continuous days and were selected with blasticidin at 2 μg/ml for 3 days. The resistant cells were harvested for qRT-PCR, or fixed for Alkaline phosphatase (AP) staining and immunofluorescence staining. AP staining was performed with AP staining kit from System Biosciences.

### Protein Analysis

Cells were lysed in 2% SDS lysis buffer (2% SDS, 50 mM Tris-HCl [pH 6.8], 10% glycerol). Proteins were separated by SDS-PAGE and analyzed by Western blotting. Signals from horseradish peroxidase-labelled secondary antibodies were detected with chemiluminescence detection reagents (Thermo Scientific). Signals from fluorescent-labelled secondary antibody were detected by Odyssey Infrared Imaging system (LI-COR Biosciences). p53 (DO1, Santa Cruz), phospho-p53 (Ser15) (Cell Signaling), acetyl-p53(K382) (Cell Signaling), acetyl-p53 (K120) (10E5, Abcam), p53 mono methyl K372 (Abcam), p21(C-19, Santa Cruz), PARP (C2-10, BD Pharmingen), Caspase-3 (Cell Signaling), Cleaved Caspase-3 (Asp175) (Cell Signaling), Rb (4H1, Cell Signaling), phospho-Rb (Ser 780) (Cell Signaling), histone H3K27me3 (Millipore), EZH2 (3147, Cell Signaling), SUZ12 (D39F6, Cell Signaling), and actin (Millipore) antibodies were purchased commercially. Hybridoma cells producing MDM2 antibody (4B11) were kindly provided by Dr. Yanping Zhang (UNC, Chapel Hill, NC), and hybridoma supernatant was used for MDM2 detection. The half-life of p21 protein was measured by Western blotting using the cells treated with cycloheximide (100 μg/ml) for indicated length of time.

### Indirect Immunofluorescence

Cells cultured on cover slips or chamber slides were fixed in 4% paraformaldehyde and permeabilized with 0.1% Triton X-100, followed by blocking with 1% goat serum in PBS. Cells were incubated with primary antibody and secondary antibody (Alexa Fluors, Life Technologies) respectively for 1 hour and counterstained with Hoechst. Images were captured with a confocal microscope (LSM 510 META, Zeiss). Primary antibodies used in this study were SSEA-4 (Chemicon), TRA-1-80 (Chemicon), NANOG (R&D Systems), SOX2 (Chemicon), Lamin A/C (JOL2, Millipore), p21 (C-19, Santa Cruz), human p53 (DO-1, Santa Cruz) and mouse p53 (CM5, novocastra), Myc (A7, Thermo Fisher Scientific), OCT4 (C-10, Santa Cruz) and LMNB1 (119D5-F1, abcam).

### Quantitative Real-Time PCR

Total RNA was extracted using TRIzol (Life Technologies) and RNeasy Mini Kit (Qiagen). cDNA was synthesized using iScript cDNA synthesis kit (BioRad). Quantitative RT-PCR was performed with SYBR Green (KAPA Biosystems) using the CFX96 System (BioRad). Relative expression was calculated using *TBP*, *actin* (*ACTA1*), or *Gapdh* as an internal control by BioRad CFX manager software. The primers used are shown in the supplementary material and methods.

### Chromatin Immunoprecipitation (ChIP) Analysis

Chromatin Immunoprecipitation assay was performed as previously described with slight modifications^55^. Cells were crosslinked with 1% formaldehyde for 10 min at 37 °C and the reaction was stopped by adding glycine to a final concentration of 0.125 M for 5 min at room temperature. After washing with PBS, cells were lysed in Lysis Buffer A (50 mM Tris-HCl [pH 8.0], 10 mM EDTA, 1% SDS) and sonicated for 90 min (30 second on/30 second off) using Diagenode Bioruptor Standard (Model UCD200). Samples were centrifuged for 10 min at 16,000 x g at 4 °C. Supernatant was diluted 10 times with dilution buffer (0.01% SDS, 1.1% Triton X-100, 1.2 mM EDTA, 16.7 mM Tris-HCl [pH 8.0], 167 mM NaCl, protease inhibitor cocktails, 1 mM PMSF, 1 mM Na_3_VO_4_), and was precleared with Protein G Agarose with salmon sperm DNA (Millipore) for 60 min at 4 °C, following the spin at 800 x g for 2 min at 4 °C. Supernatant was collected and incubated with antibodies overnight at 4 °C. The immunocomplex was recovered by 2 hrs incubation with Protein G Agarose/salmon sperm DNA at 4 °C. The agarose beads were washed for 10 min sequentially with the following buffers at 4 °C: TSE I (0.1% SDS, 1% Triton X-100, 2 mM EDTA, 150 mM NaCl, 20 mM Tris-HCl [pH 8.0]), TSE II (0.1% SDS, 1% Triton X-100, 2 mM EDTA, 500 mM NaCl, 20 mM Tris-HCl [pH 8.0]), TSE III (0.25 M LiCl, 1% NP-40, 1% deoxycholate, 1 mM EDTA), and twice with TE (10 mM Tris-HCl [pH 8.0], 1 mM EDTA). The bound DNA was eluted 3 times with 75 μL elution buffer (1% SDS, 0.1 M NaHCO_3_, 1 mM DTT) and pooled. Crosslinking was reversed by adding 9 μL 5 M NaCl and incubating at 65 °C overnight. After Proteinase K and RNAse A treatment, DNA was recovered by a QIAQuick PCR Purification Kit (Qiagen) and used as a template. PCR was carried out with SYBR Green (KAPA Biosystems) using the CFX96 System (BioRad). The antibodies and primers used are previously described^56^ and shown in the supplementary material and methods.

### Bisulfite Sequencing Analysis

Bisulfite Sequencing was performed with EpiTect Bisulfite Kit (Qiagen) according to the manufacturer’s protocol. The CpG rich promoter regions of *p21* (−131 − + 97 bp and +73 − + 370 bp in relation to the TSS) were amplified by PCR using primers previously described^57^. PCR products were subcloned into the PCR2.1-TOPO vector (Life Technologies) and individual clones were sequenced. Clones with at least 90% cytosine conversion were considered as unmethylated sites. At least 8 replicates were performed for each of the selected regions.

### Polysome fractionation

20 million cells were incubated with 100 μg/mL of cycloheximide for 10 min. Following harvesting, the cell pellets were resuspended in 150 μl RSB buffer (20 mM Tris-HCl [pH 7.4], 20 mM NaCl, 30 mM MgCl_2_, 200 μg/mL cycloheximide, 0.2 mg/mL heparin, 1000 unit/mL RNasin), and then lysed with an equal volume of lysis buffer (1X RSB, 1% Triton X-100, 2% Tween-20, 200 μg/ul heparin and 1% Na deoxycholate). Samples were kept on ice for 10 min then centrifuged at 13,000 x g for 10 min at 4 °C. Extracts were loaded onto 10% to 50% linear sucrose gradients (prepared in 10 mM Tris-HCl [pH 7.4], 75 mM KCl and 1.5 mM MgCl_2_), and centrifuged at 36,000 rpm for 90 min at 8 °C in a SW41 rotor (Beckman Coulter). A piston gradient fractionator (BioComp Instruments) was used to collect twelve fractions from the top of the gradient. The absorbance at 254 nm was measured with a UV-M II monitor (Biorad). 110 μL of 10% SDS and 12 μL of proteinase K (10 mg/mL Invitrogen) were incubated with each 1 mL fraction for 30 min at 42 °C.

RNA was purified from each fraction using Phenol Chloroform Isoamyl extraction followed by clean up on an RNeasy column (Qiagen) to remove the heparin. Superscript III Reverse Transcriptase (Invitrogen) was used to generate cDNA from equal volumes of RNA from each fraction according to the manufacturer’s instruction. Two bacterial spike-in poly-A RNAs were added before RNA purification to equal volumes of fraction. These were used as normalization controls for qRT-PCR. For qRT-PCR, SYBR Green was used with gene specific primers (listed in the supplementary material and methods) on an ABI PRISM 7900 Sequence Detection Systems.

### Data analysis of histone modification and p53 ChIP and RNAseq from public data set

The data of H3K27me3 ChIP-seq, H3K4me3 ChIP-seq, and RNAseq in H1 hESCs and H1 hMSCs were obtained from the European Nucleotide Archive (accession number: SRP000941): http://www.ebi.ac.uk/ena/data/view/SRP000941^38^. The information of H1 ESCs as well as the method of generating MSCs by differentiation is previously described^38^. ChIP seq reads^37^,^38^ were mapped using Bowtie version 0.12.8^58^. Peak calling was done with MACS 1.4.0^59^. The human genome version hg19 was used as reference. Peaks were selected based on P-value (MACS) cutoff of 200, a fold enrichment of at least 20 over the control data, and a maximum distance of 50 kb to the TSS. The protein coding genes with the nearest transcription start sites to p53 ChIP-Seq peaks^37^ with a maximum distance of 50 kb were used as p53 candidate target genes. Histone modification levels at the TSS were estimated based on the read count in a 500 bp window around the TSS. RNA-Seq data was mapped using Tophat2^60^. FPKM values were calculated using Cufflinks 2.1.1^61^. Differential expression was calculated using DESeq2^62^.

## Additional Information

**How to cite this article**: Itahana, Y. *et al*. Histone modifications and p53 binding poise the *p21* promoter for activation in human embryonic stem cells. *Sci. Rep.* **6**, 28112; doi: 10.1038/srep28112 (2016).

## Supplementary Material

Supplementary Information
