# Supplementary material for: Histone modifications and p53 binding poise the p21 promoter for activation in human embryonic stem cells
Source: Sci Rep. 2016 Jun 27;6:28112. doi: 10.1038/srep28112 (PMC4921813; doi:10.1038/srep28112)
Supplement: Supplementary Information [file srep28112-s1.pdf]

Inventory of Supplementary Information

Histone modifications and p53 binding poise the *p21* promoter for activation in human embryonic stem cells

Yoko Itahana, Jinqiu Zhang, Jonathan Göke, Leah A Vardy, Rachel Han, Kozue Iwamoto, Engin Cukuroglu, Paul Robson, Mahmoud A. Pouladi, Alan Colman, Koji Itahana

Supplementary Figures

Figure S1: Validation of H9 hESCs and H9 hMSCs using pluripotent and differentiation markers respectively.

Figure S2: p21 expression in N1-hiPSCs is very low even upon p53 activation by DNA damage.

Figure S3: Post-translational modifications of p53 in hESCs and hMSCs.

Figure S4: Translation analysis of *p21* mRNA in WI-38 cells.

Figure S5: p53 localizes in the nucleus in mouse E14 mESCs and human H9 hESCs.

Figure S6: DZNep triggers degradation of EZH2 and SUZ12.

Supplementary Tables

Table S1: Coordinates of p53 ChIP-Seq peaks in H1 hESCs.

Table S2: The comparison of H3K27me3 marks, H3K4me3 marks, and mRNA levels of p53 candidate target genes between H1 hESCs and H1 hMSCs.

Supplementary Figure Legends and Table Legends

Supplementary Materials and Methods

Primers used for Quantitative Real-Time PCR

The sequences used for RNA Interference

The antibodies and primers used for Chromatin Immunoprecipitation (ChIP) Analysis

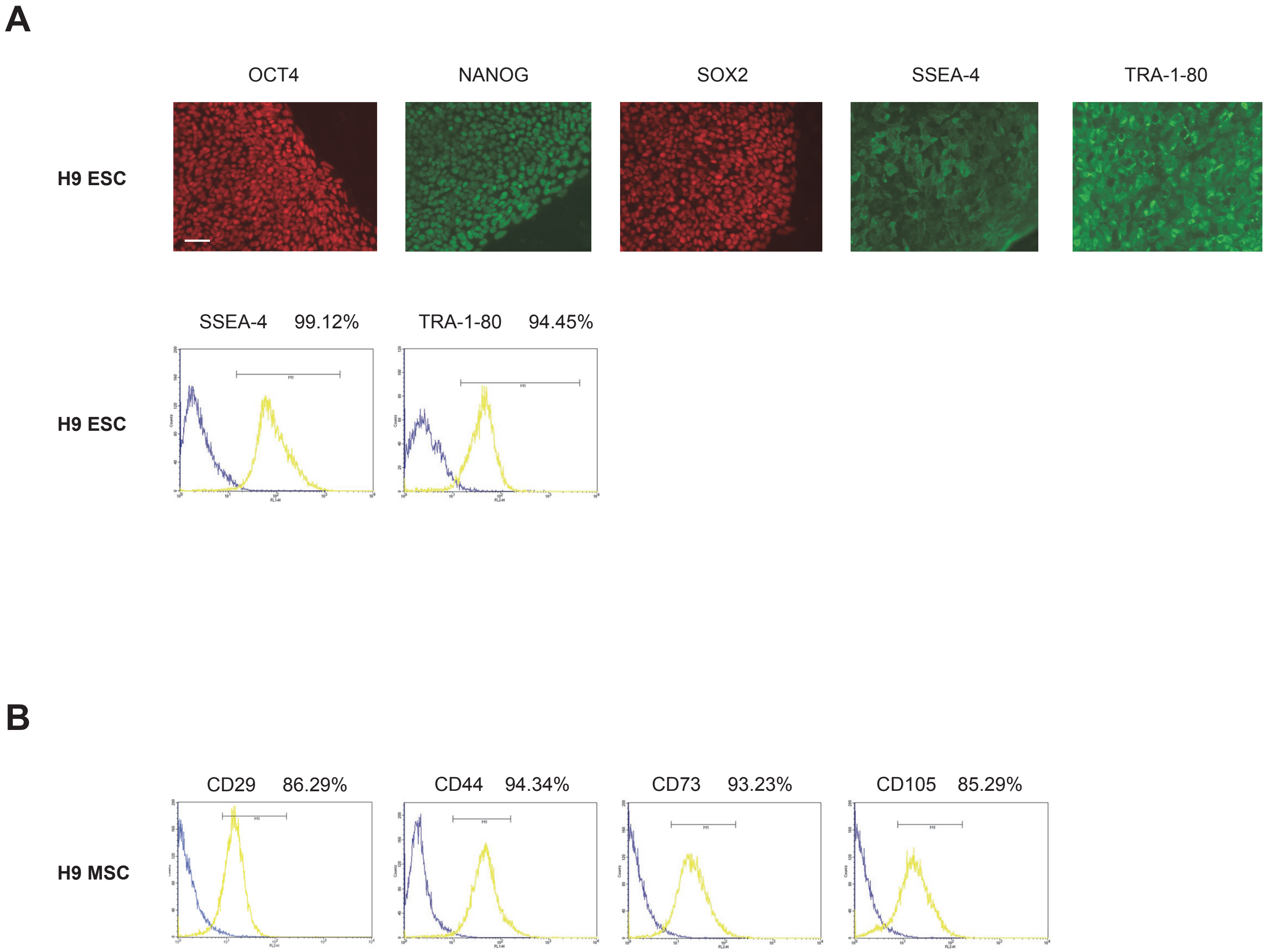

Figure S1. Validation of H9 hESCs and H9 hMSCs using pluripotent and differentiation markers respectively.

(A) Immunofluorescence analyses of H9 hESCs for pluripotent markers, OCT4, NANOG, SOX2, SSEA-4, and TRA-1-80 are shown. Expression of pluripotent cell surface markers, SSEA-4 and TRA-1-80 were also examined by flow cytometry analysis. The percentage of SSEA-4 positive and TRA-1-80 positive cells are shown.

(B) Differentiation status of H9 hMSCs was validated by flow cytometry analysis for MSC surface markers, CD29, CD44, CD73 and CD105. The percentage of positive cells were shown.

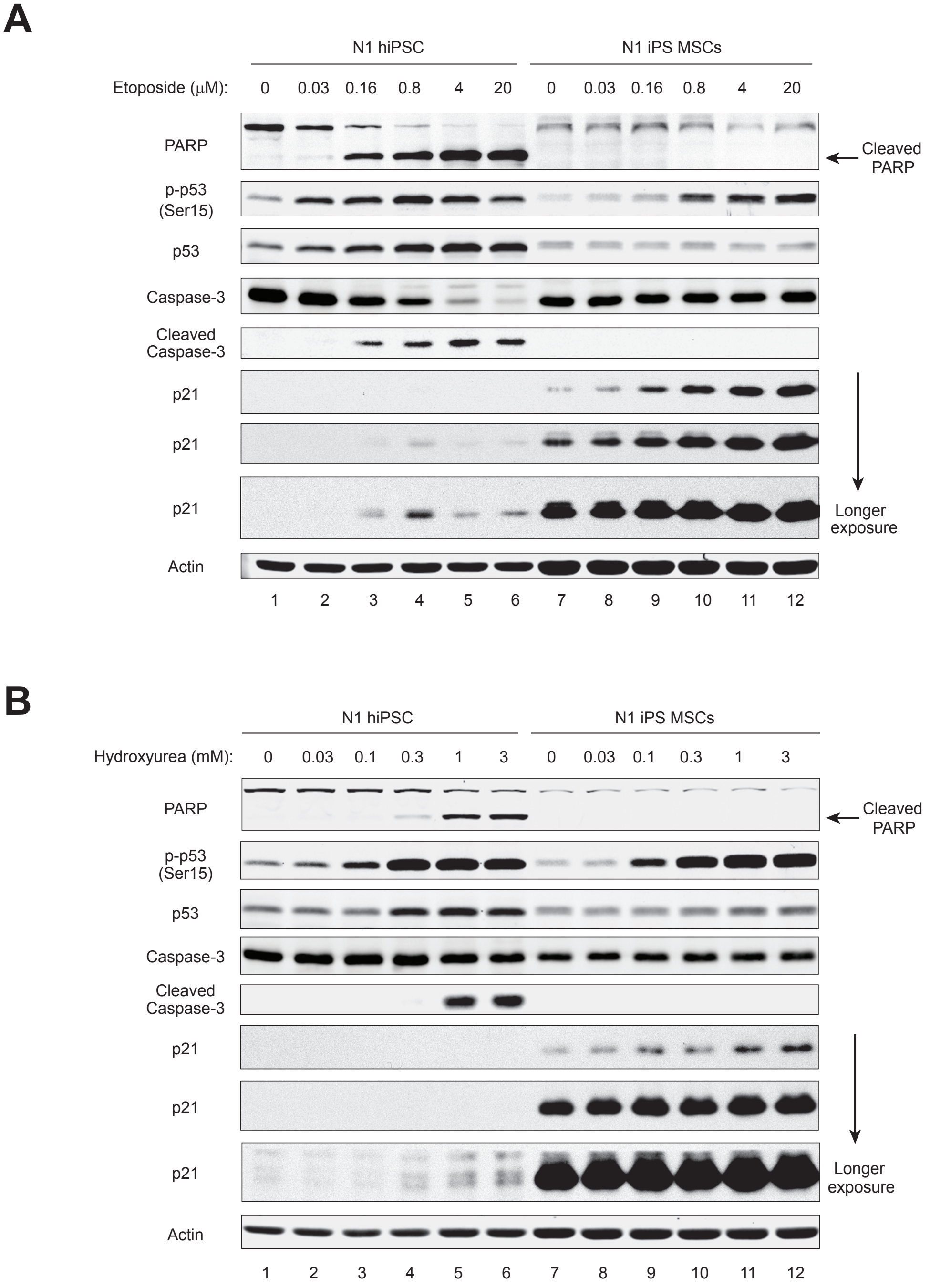

Figure S2. p21 expression in N1-hiPSCs is very low even upon p53 activation by DNA damage.

N1-hiPSCs and N1-hiPS MSCs were treated with the indicated concentration of etoposide (A) or hydroxyurea (B) for 24 hrs and harvested for Western blotting. 50 μg of protein lysate was loaded in each lane. The passage number of N1-hiPSCs is P37 (A) and P36 (B), and the passage number of N1-hiPS MSCs is P9 (A) and P8 (B).

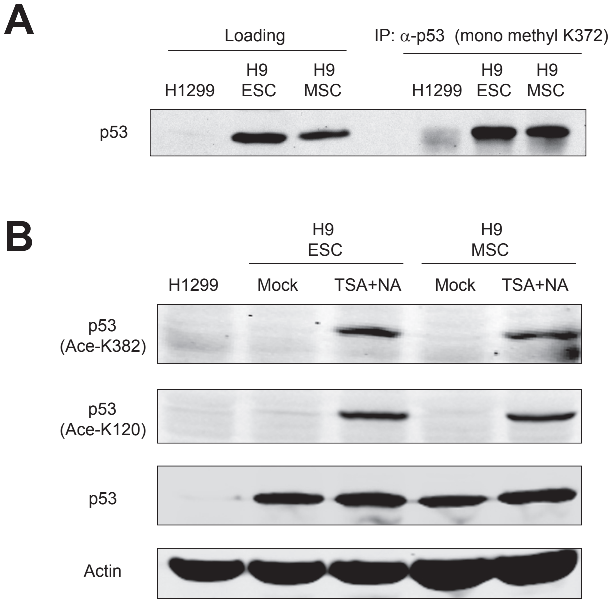

Figure S3. Post-translational modifications of p53 in hESCs and hMSCs.

(A) Detection of p53 mono-methylation at K372. Cell lysates were immunoprecipitated with antibody specific for p53 mono-methylated at K372. Immunoprecipitates were separated by SDS-PAGE and detected with p53 antibody by Western blotting analysis. The levels of mono-methylation at K372 are similar between H9 hESCs and hMSCs. H1299 (*p53*-null, human non-small cell lung carcinoma cell line) was used as a negative control. The passage numbers of H9 hESCs and hMSCS are P40 and P11 respectively.

(B) Detection of p53 acetylation at K120 and K382. Cells were treated with 0.5 μM Trichostatin A (TSA) and 5 mM Nicotinamide for 16 hours, and protein lysates were harvested for Western blotting analysis. The levels of acetylation at K120 and K382 are similar between H9 hESCs and hMSCs. The passage numbers of H9 hESCs and hMSCs are P28 and P9 respectively.

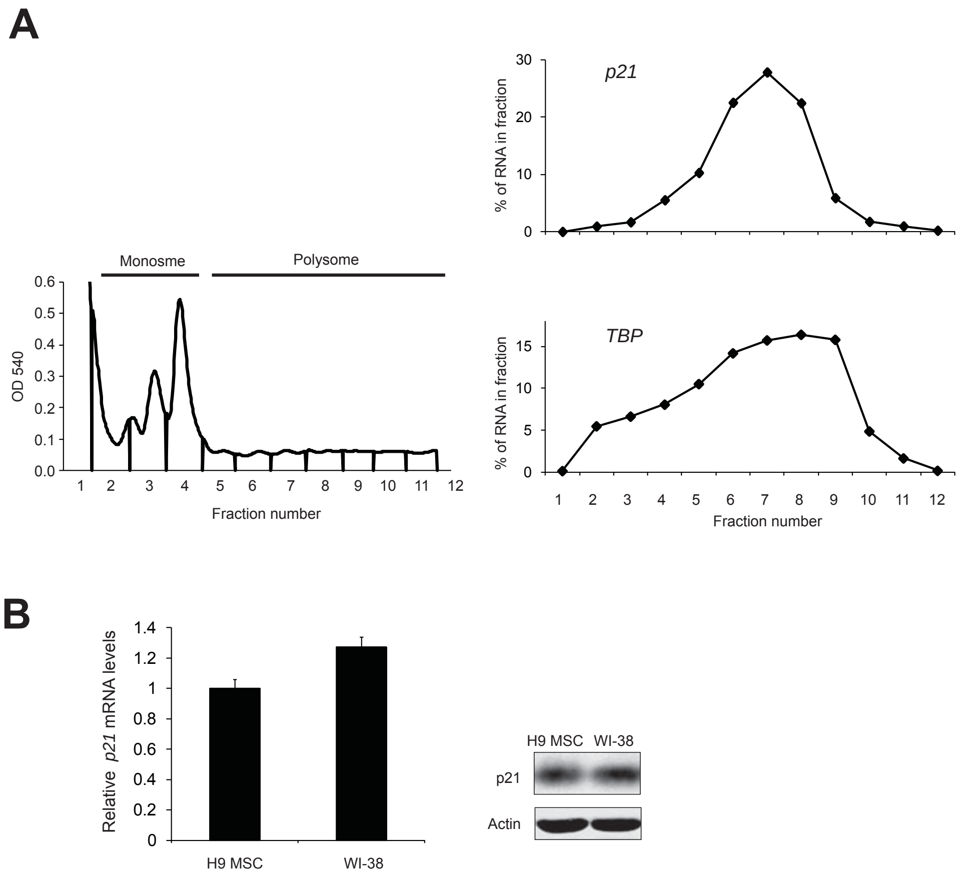

Figure S4. Translation analysis of *p21* mRNA in WI-38 cells.

(A) Translation analysis of *p21* mRNA in WI-38 normal human fibroblasts. Representative polysome profile from WI-38 cells indicating monosome and polysome fractions is shown in the left panel. qRT-PCR analysis shows the percentage of *TBP* and *p21* mRNA in each fraction in the right panels.

(B) The expression of *p21* mRNA and proteins is similar between H9 hMSC and WI-38 cells. Cell lysates from H9 hMSC and WI-38 cells were analyzed by qRT-PCR (left) and Western blotting (right). The passage number of H9 hMSCs is P6 (PCR) and P8 (WB).

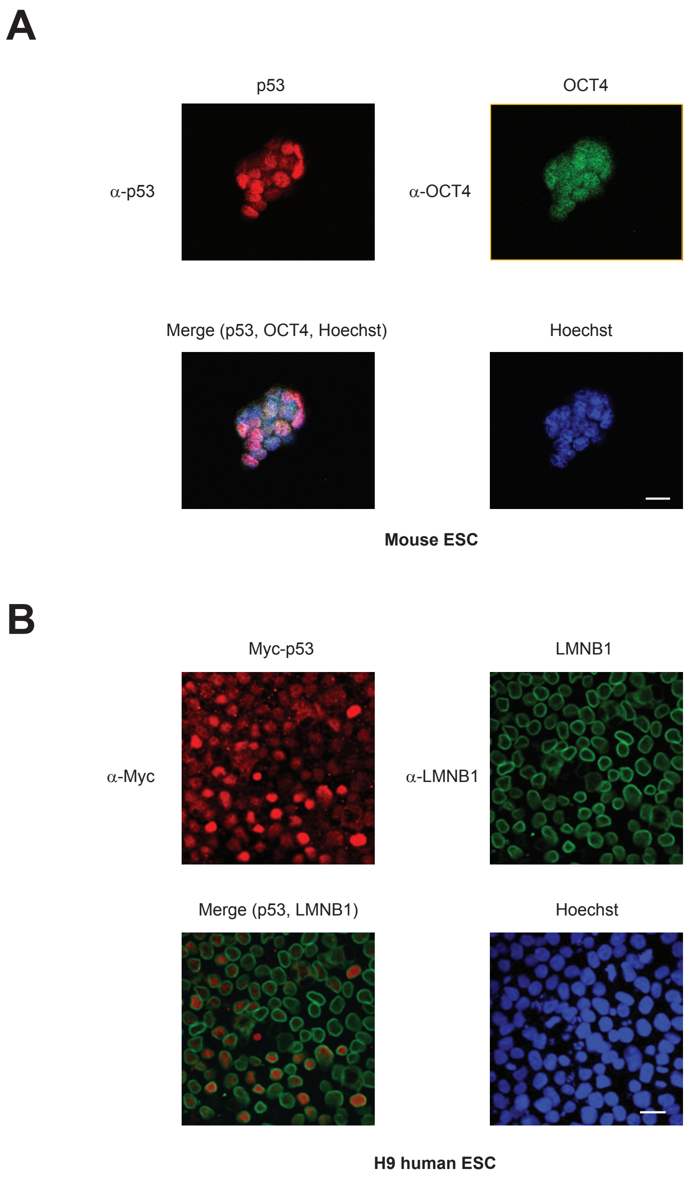

Figure S5. p53 localizes in the nucleus in mouse E14 mESCs and human H9 hESCs.

(A) Immunofluorescence assay of mouse E14 mESCs was performed with the indicated antibodies. A merged image and Hoechst staining are also shown. OCT4 is an ES cell marker that localizes in the nucleus (scale bar: 20 μm).

(B) H9 hESCs were infected with lentivirus carrying inducible pTRIPZ-myc-p53. Cells were selected with 1 μg/ml puromycin, and p53 was induced by treating cells with 1 μg/ml DOX for 3 days. Immunofluorescence assay was performed with the indicated antibodies. A merged image and Hoechst staining are also shown. Note that myc-p53 is surrounded by the nuclear envelope marker, lamin B1 (LMNB1) (scale bar: 20 μm).

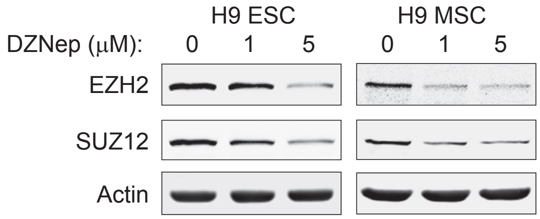

Figure S6. DZNep triggers degradation of EZH2 and SUZ12.

H9 hESCs and hMSCs were treated with DZNep at the indicated dose for 2 days. Proteins were harvested for Western blotting analysis with the indicated antibodies. The passage numbers of H9 hESCs and hMSCs are P35 and P9 respectively.

Table S1: Coordinates of p53 ChIP-Seq peaks in H1 hESCs

Gene Symbol	Chromosome No.	Start	End	p53 binding peak value	pvalue	Fold_enrichment	Strand direction	distance from TSS
OR52K2	chr11	4485230	4486189	4485689	2170.2	250.3	+	15164
CLEC2A	chr12	10063376	10064064	10063838	1921.1	152.39	-	21107
IFT74	chr9	26975584	26977930	26976624	1709.45	123.48	+	59
AEN	chr15	89163622	89165028	89164559	1568.14	114.29	+	32
IFNGR2	chr21	34739307	34740083	34739708	1343.33	80.88	+	-35494
RPS27L	chr15	63448793	63450527	63449521	1318.99	84.04	-	-42
REV3L	chr6	111804253	111805612	111805338	1194.09	58.34	-	-424
C2orf51	chr2	88836959	88838708	88838011	1167.91	69.72	+	13842
KRTAP10-7	chr21	46017825	46018517	46018060	1165.18	123.91	+	-2437
FGF2	chr4	123719248	123719972	123719510	1128.34	130.62	+	-28353
DDB2	chr11	47235948	47237417	47236552	1122.91	60.72	+	59
C1orf88	chr1	111906976	111907875	111907576	1099.26	117.86	+	18334
IER5	chr1	181103651	181104770	181104301	1097.83	86.41	+	46465
BBS9	chr7	33391216	33391936	33391487	1043	163.43	+	7268
LIF	chr22	30641224	30642713	30641870	1029.79	78.1	-	857
RPL39L	chr3	186900829	186901524	186901172	1018.26	111.12	-	-2477
BIRC8	chr19	53798134	53798734	53798530	983.04	103.9	-	-3656
BCL2A1	chr15	80295276	80295976	80295702	962.24	92.86	-	-31915
TRIAP1	chr12	120883781	120884530	120884135	950.56	84.65	-	79
BAX	chr19	49457858	49458724	49458516	872.65	31.43	+	330
CHD2	chr15	93493026	93493839	93493254	868.15	142.87	+	45551
CDKN1A	chr6	36643346	36644467	36644224	849.9	64.52	+	-1364
PLN	chr6	118895116	118895753	118895382	797.42	69.5	+	25921
PTGFRN	chr1	117420945	117422309	117421895	779.31	53.57	+	-30784
MDM2	chr12	69202435	69203100	69202704	768.06	95.75	+	-93
PLK3	chr1	45265155	45266258	45265656	735.33	37.66	+	-241
DCP1B	chr12	2113102	2114430	2113824	735.2	42.86	-	-124
BBC3	chr19	47734512	47734908	47734709	730.61	52.38	-	-259
CLRN3	chr10	129661369	129662094	129661665	709.41	95.99	-	29545
UBP1	chr3	33509260	33509866	33509503	707.55	58.17	-	-27425
RPS19	chr19	42363645	42365031	42364427	701.86	47.86	+	439
SPAG9	chr17	49031394	49032469	49031777	663.86	46.82	-	39520
COBL	chr7	51392862	51393527	51393107	641.11	59.47	-	-8593
GDF15	chr19	18483992	18485202	18484774	631.99	57.15	+	-12194
PLK2	chr5	57757888	57758306	57758101	620.8	95.24	-	-2015
SNRPN	chr15	25103850	25104423	25104045	606.36	71.43	+	2347
SCIN	chr7	12596833	12597400	12597168	594.66	113.82	+	-13144
TNFRSF10B	chr8	22925479	22927248	22926133	590.78	33.12	-	400
PIAS2	chr18	44457368	44457879	44457569	571.34	66.67	-	13638
AEBP2	chr12	19584747	19585502	19585008	568.04	48.11	+	-7600
PTP4A1	chr6	64283216	64284122	64283670	534.51	46.97	+	1195
HLA-B	chr6	31296291	31297181	31296553	532.28	75	-	28402
GNG4	chr1	235838282	235839029	235838614	522.74	62.59	-	-24561
PHLDA3	chr1	201437543	201439567	201438329	517.02	32.38	-	-18
STK17A	chr7	43616974	43617501	43617246	495.6	50.65	+	-5418
PCCA	chr13	100702315	100703025	100702818	495.05	32.27	+	-38451
EDDM3A	chr14	21219697	21220797	21220338	489.38	36.98	+	6287
CCNG1	chr5	162864088	162865196	162864933	486.68	26.53	+	313
UBC	chr12	125423110	125425696	125423357	472.79	20.89	-	-21444
PTPRM	chr18	7728212	7728891	7728537	466.88	47.36	+	-26434
TRPC4	chr13	38488935	38489487	38489284	457.88	37.35	-	-44723
TGFA	chr2	70823998	70824469	70824214	443.15	66.67	-	-43068
GNG4	chr1	235832512	235833837	235833396	437.69	41.76	-	-19343
HLA-B	chr6	31304515	31305442	31305075	432.39	58.73	-	19880
SPATA18	chr4	52917277	52918630	52918132	431.78	26.73	+	550
GAS6	chr13	114540197	114541039	114540444	430.84	36.74	-	-1428
USP34	chr2	61504688	61505294	61505090	423.7	67.64	-	2377
MFSD2A	chr1	40442435	40442787	40442610	423.52	53.78	+	21788
C6orf138	chr6	48078627	48079173	48078889	419.39	24.05	-	-42465
EDA2R	chrX	65858233	65858841	65858445	419.29	69.98	-	437
GPC3	chrX	132919992	132920801	132920570	417.09	51.43	-	-32842
CHD9	chr16	53088126	53090018	53088924	412.28	25.28	+	-21
GLT6D1	chr9	138536196	138536969	138536761	410.25	24.49	-	-5376
GPX1	chr3	49395590	49396724	49395997	408.82	29.41	-	35
CFTR	chr7	117270571	117271164	117270770	404.44	91.43	+	19099
NHLH2	chr1	116381241	116382488	116382062	396.59	22.98	-	1299
FAM65B	chr6	24985566	24986072	24985767	389.09	30.25	-	-49580
PTMA	chr2	232526510	232527719	232527373	378.93	21.35	+	-45021
TRPM1	chr15	31442401	31443662	31443138	377.72	33.93	-	10018
ACTR3B	chr7	152430401	152431011	152430826	376.42	35.72	+	-26008
CLEC3A	chr16	78025448	78026374	78025753	375.69	33.84	+	-30659

KL	chr13	33589950	33591403	33590285	374.85	40.86	+	78
HAUS4	chr14	23425606	23426592	23425777	368.78	32.14	-	453
RBM22	chr5	150080180	150081169	150080672	366.91	42.86	-	-4
C6orf97	chr6	151845080	151845554	151845352	366.43	34.78	+	30177
SACS	chr13	23973026	23973823	23973621	362.01	41.84	-	12030
SPTLC1	chr9	94909285	94910045	94909772	346.72	28.57	-	-32108
C9orf69	chr9	139001083	139002840	139001665	345.73	33.08	-	9043
SMEK1	chr14	91975662	91977378	91976804	341.76	24.53	-	17
C8orf22	chr8	50022619	50023771	50023169	340.97	41.07	+	38256
HARS	chr5	140070952	140071317	140071134	339.91	48.8	-	-13
SUSD1	chr9	114906743	114907929	114907326	333.36	56.34	-	-2581
PHIP	chr6	79787591	79788645	79788460	327.01	47.73	-	-508
ASCC3	chr6	101328621	101329620	101328965	326.37	23.81	-	221
PTPRN	chr2	220158774	220159955	220159459	325.55	25	-	1603
PTPRM	chr18	7733681	7733989	7733834	325.17	46.16	+	-21137
AP000769.1	chr11	65188219	65189857	65188796	324.96	28.57	+	-33932
CTNNA1	chr5	138044175	138045112	138044549	324.87	47.04	+	-44237
CDH1	chr16	68796461	68798128	68797480	324.65	28.57	+	26225
C1QL2	chr2	119913324	119915198	119914056	322.11	20.03	-	2408
CDKN1A	chr6	36634605	36635365	36634993	321.88	31.78	+	-10595
FOXB1	chr15	60288305	60289804	60289618	316.98	28.85	+	-6803
TRANK1	chr3	36974417	36975031	36974680	315.47	29.87	-	11867
KCND2	chr7	119913574	119914058	119913842	315.36	41.36	+	120
C9orf43	chr9	116172076	116174134	116172979	310.08	23.02	+	13
WWOX	chr16	78133225	78134297	78133910	308.54	24.18	+	234
FAM117B	chr2	203499281	203499609	203499444	304.34	50	+	-457
C2orf77	chr2	170512482	170512756	170512618	295.8	46.7	-	25215
WEE1	chr11	9631473	9631940	9631690	295.52	29.85	+	23429
MAPK1IP1L	chr14	55517598	55519123	55518189	292.88	24.68	+	-160
IRF2BP2	chr1	234754170	234754658	234754373	292.5	39.69	-	-9103
CLEC3A	chr16	78029135	78030700	78029927	292.31	21.68	+	-26485
KLRG1	chr12	9108373	9109398	9108879	291.19	46.43	+	6239
ADCY9	chr16	4190339	4190975	4190531	291.17	36.4	-	-24346
KDM1A	chr1	23345679	23346091	23345884	289.26	59.53	+	-57
PIAS2	chr18	44448337	44449024	44448497	288.63	33.62	-	22710
CALM2	chr2	47402596	47404636	47404047	287.56	22.62	-	-308
LRRC16A	chr6	25278637	25280240	25279195	285.02	22.45	+	-461
KRTAP10-2	chr21	45968798	45969582	45969099	282.4	33.62	-	2288
GPM6A	chr4	176936358	176936982	176936557	281.26	52.53	-	-12743
ZFP36L2	chr2	43444631	43446122	43445310	280.84	20.95	-	8437
GABRB3	chr15	26841021	26841413	26841216	280.52	52.75	-	33020
EFHC2	chrX	44108284	44108570	44108426	280.17	46.7	-	-23425
UBA5	chr3	132345726	132346376	132345995	279.84	38.53	+	-27295
STK32B	chr4	5135515	5136298	5135715	278.71	42.86	+	-5572
C8orf34	chr8	69542965	69543396	69543153	277.68	35.49	+	5459
FGFBP3	chr10	93668156	93668917	93668582	276.67	32.47	-	657
CGA	chr6	87788664	87789408	87788996	275.08	49.61	-	15827
ELOVL6	chr4	111118217	111120156	111119815	274.41	22.65	-	-7
THNSL2	chr2	88491048	88491493	88491269	274.36	25.72	+	20290
SLC12A8	chr3	124839091	124840004	124839841	273.66	26.79	-	-144
IGFL3	chr19	46636591	46637865	46636771	273.23	33.93	-	-8841
TLR5	chr1	223302053	223302921	223302640	269.06	33.93	-	5457
UBE2D2	chr5	138902938	138904466	138904056	268.83	21.6	+	-1960
GABBR1	chr6	29599483	29600475	29600263	268.53	24.68	-	295
FUT9	chr6	96462935	96464453	96463494	264.26	38.1	+	-366
OSTM1	chr6	108454765	108455363	108454965	263.96	34.12	-	32078
KHNYN	chr14	24898835	24900253	24899034	263.94	24.07	+	-107
PRDM1	chr6	106546233	106547306	106546433	262.73	25.72	+	-48
NEO1	chr15	73458089	73458550	73458291	261.51	33.77	+	29965
LHX8	chr1	75557732	75558256	75557926	260.63	36.74	+	-36193
NFATC4	chr14	24835448	24837638	24835735	259.26	21.43	+	-382
HES2	chr1	6474215	6475147	6474726	258.39	26.67	-	5236
ZMYND8	chr20	45947952	45949052	45948384	258.35	20.95	-	-20741
FBLN1	chr22	45865405	45866189	45865871	258.11	52.38	+	-32247
EFNA3	chr1	155056737	155058174	155057417	256.59	23.4	+	6032
KIAA0889	chr20	35463471	35464695	35463936	255.88	29.33	-	-19253
FBXO44	chr1	11710397	11711010	11710673	255.28	31.25	+	-3759
HDAC1	chr1	32756630	32758039	32757337	254.83	31.75	+	-350
RBPJ	chr4	26408005	26408850	26408443	254.69	32.66	+	-21899
MSX1	chr4	4860851	4861565	4861107	251.15	29.76	+	-286
FAM151B	chr5	79782886	79784646	79784188	250.71	23.9	+	400
IQGAP2	chr5	75698020	75699656	75698952	250.54	25.51	+	-122
ATXN7L1	chr7	105516451	105517290	105516780	250.31	32.14	-	142

KIAA1671	chr22	25457701	25458019	25457859	248.79	27.27	+	-8077
P2RX4	chr12	121622165	121622557	121622360	247.5	57.15	+	-25300
CDC14A	chr1	100816905	100819057	100817643	245.49	21.29	+	353
SNX12	chrX	70287973	70288969	70288314	244.9	30.16	-	-42
SP7	chr12	53738619	53739894	53739053	244.14	21.43	-	45
HYAL2	chr3	50358122	50359778	50359469	243.52	23.58	-	-326
RSBN1L	chr7	77324813	77326208	77325392	243	22.08	+	-368
LDB1	chr10	103879541	103881491	103880385	242.1	23.44	-	-176
PITX2	chr4	111535271	111535950	111535501	241.13	27.98	-	6370
IPP	chr1	46216078	46217092	46216561	239.94	22.56	-	-240
SPECC1	chr17	20036889	20038127	20037079	239.85	40.86	+	-22306
LRRC20	chr10	72141563	72142878	72142254	239.35	25.72	-	90
DENND2D	chr1	111756854	111757759	111757285	238.41	34.53	-	-10255
PRIMA1	chr14	94218504	94218936	94218733	237.11	30.53	-	35371
FMN1	chr15	33191373	33191882	33191676	237	36.74	-	2564
UBE2F	chr2	238848275	238849207	238848689	236.77	35.72	+	-26908
FBXO31	chr16	87417400	87418632	87417807	235.95	21.98	-	-414
MOCS2	chr5	52405248	52406107	52405439	235.86	28.57	-	129
BRWD3	chrX	80065344	80065907	80065579	235.33	40.86	-	-393
RGS9	chr17	63132966	63133875	63133162	234.79	36.28	+	-294
INO80	chr15	41457703	41458021	41457861	234.39	46.7	-	-49310
USP25	chr21	17101792	17102049	17101920	234.39	37.94	+	-424
TIFAB	chr5	134793243	134793587	134793414	234.3	49.57	-	-5326
LECT2	chr5	135265642	135267069	135266823	233.9	23.8	-	22003
ELOVL5	chr6	53213653	53214416	53214034	233.9	22.74	-	-88
OSBPL8	chr12	77001797	77002659	77002097	233.85	31.87	-	-48509
GEMIN8	chrX	14047556	14048201	14048031	233.76	42.86	-	-20
GAS7	chr17	10108894	10109521	10109125	233.7	28.2	-	-7258
TET1	chr10	70322774	70323542	70323111	233.44	37.47	+	2698
RUNX1T1	chr8	93114789	93115996	93115574	232.92	25.72	-	-61
PRMT1	chr19	50179389	50179955	50179554	232.6	24.16	+	56
RFC4	chr3	186512926	186513165	186513045	232.52	34.29	-	-3407
PDHX	chr11	35039485	35039760	35039622	232.08	25.4	+	40291
DLG1	chr3	196969577	196970407	196970134	231.74	22.08	-	17197
FAM101A	chr12	124758060	124758915	124758321	231.3	34.54	+	-15389
POLH	chr6	43542848	43544242	43543888	230.66	20.54	+	1
GCC2	chr2	109061639	109062174	109061969	230.35	31.75	+	-3048
ALPL	chr1	21835945	21837623	21836857	230.09	24.68	+	992
WNK3	chrX	54384274	54385419	54384797	229.84	27.21	-	277
DNMT3B	chr20	31349207	31349924	31349621	228.93	27.47	+	-570
ABLIM2	chr4	8065058	8065831	8065614	228.48	27.16	-	7939
EIF4G3	chr1	21503081	21504013	21503499	227.46	25.8	-	-123
KCNQ3	chr8	133203904	133204593	133204408	227.2	51.43	-	-4949
LARP1	chr5	154091841	154092719	154092280	226.41	30	+	-182
ZNF598	chr16	2059651	2060757	2060007	225.81	35.72	-	-245
PIAS1	chr15	68346231	68347205	68346770	225.54	42.86	+	-144
MEIS2	chr15	37394596	37395176	37394802	224.78	31.14	-	-1299
ANK1	chr8	41676201	41676838	41676457	224.31	22.45	-	-21318
KRCC1	chr2	88323798	88325279	88324339	224.23	23.38	-	30908
PDCD5	chr19	33061060	33061889	33061245	223.64	35.02	+	-10851
PINX1	chr8	10697049	10697672	10697501	223.32	22.78	-	-116
MED4	chr13	48646158	48647061	48646623	223.2	44.9	-	21118
SHMT1	chr17	18266390	18267253	18266933	223	22.45	-	-102
AC004696.1	chr19	56988690	56989836	56989362	222.97	28.57	+	-190
LAGE3	chrX	153707098	153707933	153707425	222.49	23.89	-	-158
WNT11	chr11	75919873	75920580	75920098	222.42	20.09	-	1681
KAZN	chr1	15233966	15234322	15234143	221.22	30	+	-16483
SCML2	chrX	18373038	18373521	18373236	221.04	46.7	-	-390
CHD2	chr15	93573742	93574575	93574183	220.95	23.04	+	22087
CPNE5	chr6	36761299	36762028	36761830	220.91	30.36	-	-36645
AMOTL2	chr3	134060220	134060565	134060392	220.52	38.38	-	30361
SEPT3	chr22	42381860	42382093	42381976	220.52	25.59	+	8982
ATP5J	chr21	27107013	27107887	27107455	220.49	21.43	-	-112
C10orf32	chr10	104613799	104614280	104614005	219.94	31.17	+	-14
PTPN14	chr1	214612710	214613501	214613047	219.7	29.42	-	25098
DYNC2H1	chr11	103193670	103193964	103193816	219.52	40.86	+	-35155
FNDC3B	chr3	171772130	171772383	171772256	219.52	35.02	+	7553
AC084082.3	chr8	67002390	67002647	67002518	219.52	35.02	+	-23004
CCDC68	chr18	52625298	52625536	52625416	219.52	32.1	-	-138
SPOCK2	chr10	73808220	73808796	73808419	218.55	40.54	-	39666
FTH1	chr11	61765835	61767049	61766674	218.48	29.76	-	-31543
SNTG1	chr8	50823434	50824307	50824039	218.34	52.53	+	-194
CTNND1	chr11	57555876	57556604	57556072	217.61	38.57	+	-2959

PLAG1	chr8	57123068	57123828	57123210	217.49	31.43	-	627
PPFIA3	chr19	49627908	49628975	49628466	217.48	20.78	+	-3118
MED4	chr13	48640638	48641391	48640808	217.25	35.72	-	26933
CAMK2N1	chr1	20812005	20813804	20812571	216.64	28.57	-	141
STK24	chr13	99227747	99228798	99228244	216.51	30.22	-	872
ATAD5	chr17	29158804	29159473	29158998	216.3	23.38	+	10
PIM1	chr6	37105404	37106065	37105601	216.03	34.43	+	-32378
CACNG3	chr16	24264352	24264966	24264553	215.92	31.17	+	-2321
C1GALT1	chr7	7222255	7222946	7222468	215.21	37.5	+	222
TOX3	chr16	52582294	52582784	52582479	215.03	31.75	-	-766
OR6B3	chr2	240994522	240994807	240994664	215.02	32.79	-	-9176
LTN1	chr21	30374791	30375580	30375032	214.51	24.45	-	-9756
CIRH1A	chr16	69184068	69184895	69184463	214.33	44.95	+	17930
RP11-677O4.1	chr18	11655353	11655851	11655513	212.86	28.57	-	-15816
C14orf101	chr14	57132978	57133733	57133182	212.56	43.2	+	29948
RSL1D1	chr16	11944963	11946301	11946099	211.47	25.21	-	-658
MTHFD1L	chr6	151177973	151178659	151178392	211.43	22.43	+	-8293
SGMS2	chr4	108745217	108746975	108746684	211.04	23.81	+	320
LIMCH1	chr4	41365016	41365949	41365199	210.23	21.35	+	2367
PAPPA	chr9	118919453	118920265	118919653	208.75	30.36	+	3570
MFHAS1	chr8	8750859	8751695	8751377	208.21	21.43	-	-223
SMAD2	chr18	45457826	45458617	45458036	207.7	21.43	-	-522
PSMF1	chr20	1098680	1099625	1099102	207.04	27.03	+	-139
LALBA	chr12	48995927	48996556	48996153	206.42	24	-	-32305
CHERP	chr19	16653207	16653760	16653419	206.29	25	-	-79
RPL37A	chr2	217362971	217364079	217363558	205.97	36.17	+	-20
MME	chr3	154796376	154797341	154796941	205.95	40	+	-495
PLAC8	chr4	84092097	84092568	84092301	205.48	35.72	-	-34074
HSPB1	chr7	75931739	75933308	75932854	205.14	27.47	+	-40
TMSB4X	chrX	12996329	12996880	12996693	205.12	23.38	+	2916
FAM156A	chrX	52963521	52964599	52964270	205	21.84	-	21258
SLC26A5	chr7	103097860	103098196	103098027	204.86	43.78	-	-11404
SNRPN	chr15	25093395	25093659	25093526	204.86	35.02	+	-8172
SULF1	chr8	70341362	70341632	70341496	204.86	35.02	+	-37363
C6orf126	chr6	35744039	35744954	35744244	204.69	26.46	+	-148
FSD1L	chr9	108177588	108177859	108177723	204.33	25	+	-32354
CXCL6	chr4	74702019	74703005	74702384	204.28	23.81	+	27
CALN1	chr7	71801596	71803011	71802271	203.46	21.87	-	-292
ACTA2	chr10	90750546	90751429	90751107	203.21	28.57	-	-12
ZNF407	chr18	72335400	72335670	72335534	203.17	34.29	+	-7385
ANKRD44	chr2	198065036	198065311	198065173	203.17	34.29	-	-2412
TMEM63B	chr6	44094604	44095335	44094772	203.07	26.32	+	121
UBAP2	chr9	34074073	34074557	34074341	202.91	25	-	-25395
C9orf25	chr9	34456986	34458146	34457632	202.84	34.02	-	917
CTNND1	chr11	57528776	57529647	57529375	202.69	33.22	+	85
LDHB	chr12	21810241	21811201	21810786	202.69	24.41	-	-11
EGR2	chr10	64620667	64620927	64620796	202.48	28.57	-	-41870
MAP4K4	chr2	102320318	102320876	102320674	202.11	40.82	+	5682
FBXL3	chr13	77605590	77605863	77605726	201.6	33.62	-	-4397
VTA1	chr6	142468016	142468577	142468204	201.6	40.82	+	-163
CADM2	chr3	85007597	85008852	85008258	201.44	20.95	+	126
SPRYD4	chr12	56861928	56862548	56862356	200.51	21.43	+	5
OSBPL1A	chr18	21929668	21929895	21929781	200.05	27.47	-	-38312

Table S2: The comparison of H3K27me3 marks, H3K4me3 marks and mRNA levels of p53 candidate target genes between H1 hESCs and H1 hMSCs

Selected top 10 genes in Figure 5C excluding p21 (CDKN1A) are shown in red

Gene symbol	chromosome No.	Start	Strand direction	H3K27me3 (H1)	H3K27me3 (H1 MSC)	H3K4me3 (H1)	H3K4me3 (H1MSC)	H3K27me3 ratio (ESC/MSC)	Mean RNA-Seq read count (H1)	Mean RNA-Seq read count (H1MSC)
SGMS2	chr4	108745719	+	2521	225	566	649	11.20444444	48	4599
CDKN1A	chr6	36646435	+	1706	160	1207	1606	10.6625	2292.5	112786.5
PAPPA	chr9	118916083	+	1826	205	864	627	8.907317073	411	94999
STK32B	chr4	5053169	+	2220	292	846	677	7.602739726	348.5	1590
PTPRM	chr18	7566780	+	948	160	1334	1748	5.925	2045.5	17220
TRANK1	chr3	36986547	-	1819	347	921	379	5.242074928	716	2686.5
GAS6	chr13	114567045	-	989	195	711	866	5.071794872	1872	27373.5
MEIS2	chr15	37392703	-	1135	234	1450	1279	4.85042735	19.5	14971
IER5	chr1	181057638	+	852	190	1414	1293	4.484210526	1019.5	4216
PHLDA3	chr1	201438364	-	1364	307	1754	1495	4.442996743	1787	8022.5
SUSD1	chr9	114937555	-	1046	260	935	369	4.023076923	1329	5292
ANKRD44	chr2	198175510	-	1185	308	924	663	3.847402597	72.5	1101
AP000769.1	chr11	65222728	+	1026	274	192	91	3.744525547	NA	NA
ACTA2	chr10	90751044	-	839	236	1337	713	3.555084746	273	44924.5
REV3L	chr6	111804914	-	391	114	2661	1288	3.429824561	2136	7077
WNT11	chr11	75921802	-	5968	1900	664	154	3.141052632	16.5	1
MSX1	chr4	4861393	+	5169	1656	148	347	3.121376812	36.5	685.5
KL	chr13	33590207	+	3236	1038	354	163	3.117533719	4.5	19.5
ADCY9	chr16	4166185	-	790	269	802	485	2.936802974	5191.5	12891.5
LIMCH1	chr4	41361624	+	3374	1182	852	371	2.854483926	710	14704.5
CAMK2N1	chr1	20812712	-	1407	519	1736	1232	2.710982659	1278	15427
DENND2D	chr1	111747030	-	1823	679	154	169	2.684830633	84.5	137.5
PITX2	chr4	111541871	-	6442	2416	319	117	2.666390728	80.5	3821.5
EGR2	chr10	64576125	-	2833	1162	1443	433	2.438037866	128.5	106.5
SPOCK2	chr10	73848085	-	3480	1518	544	105	2.292490119	860	118.5
AMOTL2	chr3	134093286	-	640	285	2328	1113	2.245614035	6170	6559
CPNE5	chr6	36807777	-	3453	1620	748	181	2.131481481	62.5	40.5
CXCL6	chr4	74702357	+	263	123	524	289	2.138211382	705.5	950.5
HES2	chr1	6484729	-	4296	2219	167	117	1.93600721	78	5.5
HSPB1	chr7	75932894	+	472	248	847	696	1.903225806	8982	54083
SCIN	chr7	12610312	+	1553	828	509	160	1.875603865	161	1.5
ATXN7L1	chr7	105516922	-	611	326	1639	513	1.874233129	8855.5	1972
PLAC8	chr4	84030995	-	410	219	606	245	1.872146119	44	2308
PRDM1	chr6	106546481	+	505	272	283	42	1.856617647	123.5	3188
C1QL2	chr2	119916464	-	3544	1922	668	92	1.843912591	61	0
SPATA18	chr4	52917582	+	469	261	681	454	1.796934866	343.5	4117
SNTG1	chr8	50822349	+	506	289	847	176	1.750865052	70	4.5
KCNQ3	chr8	133493199	-	3243	1859	777	150	1.744486283	1068	3046
FOXB1	chr15	60296421	+	3679	2164	1452	124	1.700092421	176.5	0
GPM6A	chr4	176923482	-	633	379	1102	321	1.670184697	1110.5	28.5
SCML2	chrX	18372846	-	442	266	537	344	1.661654135	2626.5	90.5
PHIP	chr6	79787952	-	170	104	3116	781	1.634615385	18899.5	6985
PTMA	chr2	232573246	+	234	145	6730	1749	1.613793103	280156	100903
HLA-B	chr6	31324963	-	1422	892	1214	601	1.594170404	7355.5	55022.5
SP7	chr12	53739098	-	654	412	459	200	1.587378641	15	0.5
MFSD2A	chr1	40420822	+	685	434	712	275	1.578341014	5703.5	125
EIF4G3	chr1	21503376	-	338	217	1371	653	1.557603687	19010	31177.5
TRPC4	chr13	38443859	-	382	247	2537	341	1.546558704	2583.5	985.5
PTPN14	chr1	214724565	-	265	174	2874	1318	1.522988506	20366	20887.5
ELOVL5	chr6	53213586	-	281	185	2151	921	1.518918919	47562	27504.5
OSTM1	chr6	108395940	-	363	245	1085	719	1.481632653	4184.5	14134.5
KHNYN	chr14	24899144	+	424	300	730	627	1.413333333	5954.5	12413
SLC12A8	chr3	124931608	-	433	307	658	350	1.410423453	1813	12506.5
GDF15	chr19	18496968	+	360	275	93	240	1.309090909	1182.5	36102
MFHAS1	chr8	8751154	-	454	348	1732	780	1.304597701	8441	3130
PLK2	chr5	57756086	-	218	172	1285	1008	1.26744186	2172	26602.5
C8orf34	chr8	69243460	+	625	496	1288	595	1.260080645	220	27
BBC3	chr19	47734450	-	395	314	2011	1352	1.257961783	918.5	1076.5
STK17A	chr7	43622664	+	430	342	1237	938	1.257309942	2215	6124.5
LHX8	chr1	75600567	+	2754	2258	989	79	1.219663419	166.5	1.5
LIF	chr22	30642727	-	301	247	668	654	1.218623482	597.5	3164.5
CACNG3	chr16	24266874	+	626	529	134	38	1.183364839	4.5	2
AEBP2	chr12	19592633	+	175	152	4057	1305	1.151315789	5331.5	4656.5
FGF2	chr4	123747863	+	221	193	3776	912	1.14507772	22346	6260
CCDC68	chr18	52626738	-	194	173	213	143	1.121387283	144	38313.5
PIM1	chr6	37137979	+	160	146	3555	1221	1.095890411	10367	3089.5
USP25	chr21	17102496	+	104	95	2176	827	1.094736842	7871	9720.5
ANK1	chr8	41754279	-	1695	1554	623	110	1.090733591	1514	401
CFTR	chr7	117119358	+	595	554	742	192	1.07400722	202	254
LRRC20	chr10	72142379	-	456	442	803	446	1.031674208	3518.5	3135
TNFRSF10B	chr8	22926543	-	257	255	2452	1079	1.007843137	21175.5	38464
TMEM63B	chr6	44095291	+	202	205	2006	1034	0.985365854	13710.5	7342.5
PLK3	chr1	45265897	+	260	264	849	702	0.984848485	859.5	5152
RSBN1L	chr7	77325760	+	175	179	3629	1188	0.977653631	2989	2773
CALN1	chr7	71801979	-	1113	1141	1721	63	0.975460123	2554	0.5
P2RX4	chr12	121647954	+	343	354	874	406	0.968926554	959.5	4632.5
C9orf69	chr9	139010708	-	153	158	1482	1033	0.96835443	4308	4192
EFNA3	chr1	155036224	+	333	347	1663	507	0.959654179	1369.5	172
ZFP36L2	chr2	43453747	-	150	157	4242	1471	0.955414013	16726.5	29768.5
TGFA	chr2	70781146	-	1317	1391	1148	314	0.946800863	755	1
MOCS2	chr5	52405600	-	152	161	995	451	0.944099379	1345.5	5232
PDCD5	chr19	33072163	+	274	292	1395	710	0.938356164	6585.5	12629
WWOX	chr16	78133591	+	179	191	1265	606	0.937172775	598	872.5
PTGFRN	chr1	117452679	+	245	264	1628	703	0.928030303	11178.5	20476
LAGE3	chrX	153707595	-	112	121	394	117	0.925619835	3172	1951.5
IFNGR2	chr21	34775772	+	168	183	1145	646	0.918032787	3573	9399
LARP1	chr5	154135135	+	142	159	3489	1655	0.893081761	66567.5	58622.5
RGS9	chr17	63133566	+	551	621	256	131	0.887278583	650	824.5
PRIMA1	chr14	94254826	-	1384	1588	988	77	0.871536524	997	0
C1GALT1	chr7	7222178	+	152	180	2665	760	0.844444444	19653.5	5465
FBXO31	chr16	87417362	-	126	150	1639	867	0.84	1828	3655.5
RPS27L	chr15	63449697	-	117	140	2415	553	0.835714286	5274	17218
UBA5	chr3	132379204	+	164	198	3014	974	0.828282828	3933.5	5187.5
SMEK1	chr14	91976821	-	141	171	3893	1294	0.824561404	11755	10919.5
SACS	chr13	24007840	-	223	271	1054	360	0.822878229	17295	5998.5
SNX12	chrX	70288272	-	64	78	1108	241	0.820512821	5061.5	10363.5
TMSB4X	chrX	12993363	+	106	130	1846	439	0.815384615	111472	395579.5
KCND2	chr7	119913722	+	535	658	1286	400	0.813069909	2997	236.5
WEE1	chr11	9595228	+	127	157	3912	1236	0.808917197	10569.5	1899
FBLN1	chr22	45898763	+	159	200	1481	745	0.795	46334.5	43121.5
PIAS1	chr15	68346914	+	148	187	1503	613	0.79144385	4323.5	5801.5
POLH	chr6	43543887	+	137	176	3027	1118	0.778409091	1779.5	6017
RBPJ	chr4	26322185	+	126	163	3667	1109	0.773006135	44940.5	23534.5
IRF2BP2	chr1	234745270	-	116	151	2858	1224	0.768211921	6342	22036
FNDC3B	chr3	171757418	+	112	146	2062	983	0.767123288	5270	58046.5
ZNF598	chr16	2059823	-	164	214	1739	714	0.76635514	6066.5	5865.5
CALM2	chr2	47403649	-	163	214	2712	806	0.761682243	18303.5	63243
PRMT1	chr19	50179498	+	130	171	2475	832	0.760233918	28491.5	11354
FBXO44	chr1	11714432	+	321	424	1271	608	0.757075472	2946.5	2319.5
FAM117B	chr2	203499901	+	116	154	2071	905	0.753246753	4157.5	2627
SPAG9	chr17	49124238	-	62	83	337	27	0.746987952	8367.5	16746.5
MTHFD1L	chr6	151187642	+	132	178	2008	856	0.741573034	7890.5	6574.5
UBE2D2	chr5	138940751	+	132	179	2580	804	0.737430168	11067.5	15851
MAPK1IP1L	chr14	55518349	+	160	217	2573	671	0.737327189	15679.5	22331.5
HYAL2	chr3	50358647	-	193	264	1884	552	0.731060606	11176	6763
DLG1	chr3	197025446	-	146	200	2572	925	0.73	6861.5	10887
RPL39L	chr3	186857271	-	159	222	782	341	0.716216216	2896	805.5
SPECC1	chr17	20059302	+	271	380	1383	650	0.713157895	12995	8013
LDHB	chr12	21810775	-	138	194	2550	681	0.711340206	72886	61411.5
ABLIM2	chr4	8160558	-	1190	1698	704	413	0.700824499	178	8.5

MME	chr3	154797883	+	212	303	1592	471	0.699669967	10416	34426
DCP1B	chr12	2113676	-	97	139	1966	583	0.697841727	3016	5223.5
UBE2F	chr2	238875597	+	216	319	1382	819	0.677115987	1733	2160.5
PTP4A1	chr6	64282586	+	102	151	3462	1254	0.675496689	13761.5	44093
KDM1A	chr1	23345941	+	120	178	3341	825	0.674157303	33369	12096
ZNF407	chr18	72265106	+	120	178	2361	1030	0.674157303	977	2324
FSD1L	chr9	108210315	+	157	237	1728	785	0.662447257	387	694.5
SMAD2	chr18	45457514	-	127	192	3014	860	0.661458333	8446.5	12782
ALPL	chr1	21835865	+	479	724	1322	176	0.66160221	44096.5	2597.5
C10orf32	chr10	104614019	+	96	146	1003	263	0.657534247	415	2851.5
TET1	chr10	70320413	+	190	289	3282	686	0.657439446	24068	2050
NEO1	chr15	73344051	+	96	147	2293	915	0.653061224	22459.5	40533.5
UBAP2	chr9	34048926	-	140	217	2578	895	0.64516129	13307.5	8527
RSL1D1	chr16	11945372	-	124	194	3111	896	0.639175258	34235	21321.5
SHMT1	chr17	18266808	-	121	190	1156	533	0.636842105	4112	1321
SEPT3	chr22	42372994	+	1429	2255	825	130	0.633702882	6433.5	389.5
MDM2	chr12	69202053	+	126	202	2210	748	0.623762376	13122.5	24747.5
CHD9	chr16	53242363	+	65	106	525	45	0.613207547	6949.5	12253.5
BRWD3	chrX	80065186	-	64	105	1280	299	0.60952381	11996	5104.5
OSBPL8	chr12	76953588	-	105	173	2253	1088	0.606936416	16414	18138.5
SULF1	chr8	70405028	+	299	497	81	72	0.601609658	8570	170362.5
CIRH1A	chr16	69166516	+	123	207	3261	1044	0.594202899	8052	4759
MAP4K4	chr2	102313312	+	179	301	1785	665	0.594684385	22614.5	57662
IFT74	chr9	26947037	+	105	179	2859	771	0.586592179	2400	3151
AEN	chr15	89164527	+	152	259	2449	734	0.586872587	13604.5	7598.5
FGFBP3	chr10	93669239	-	114	195	2153	560	0.584615385	36886	808
ZMYND8	chr20	45985566	-	152	261	168	51	0.582375479	25803	9235
NFATC4	chr14	24838205	+	137	236	1203	565	0.580508475	3490	8921.5
GABBR1	chr6	29595814	-	122	212	1509	493	0.575471698	11570	5293.5
ATAD5	chr17	29158988	+	129	225	2109	537	0.573333333	7934	624.5
GEMIN8	chrX	14048010	-	49	86	618	166	0.569767442	1574.5	1445.5
STK24	chr13	99229116	-	99	175	2386	990	0.565714286	21422	31420.5
TOX3	chr16	52580919	-	552	990	2490	344	0.557575758	1839	3.5
ELOVL6	chr4	111119831	-	132	240	2807	1097	0.55	28093.5	15030.5
USP34	chr2	61697903	-	95	173	2815	783	0.549132948	17470.5	18974
OSBPL1A	chr18	21977751	-	115	210	1797	468	0.547619048	10644.5	8208.5
PDHX	chr11	34938119	+	122	227	2405	743	0.537444934	4200.5	6045.5
GCC2	chr2	109065684	+	110	205	1545	546	0.536585366	11311.5	12636
SPTLC1	chr9	94877657	-	117	220	1637	453	0.531818182	18835.5	14653.5
BAX	chr19	49458134	+	118	223	1081	471	0.529147982	6147	7387
CDC14A	chr1	100817290	+	119	225	2706	832	0.528888889	1297.5	574.5
KAZN	chr1	14925213	+	846	1595	1371	126	0.530407524	1776.5	10730.5
FAM151B	chr5	79783788	+	122	231	562	224	0.528138528	90.5	484
RBM22	chr5	150080668	-	118	225	2317	709	0.524444444	4939.5	6932
UBP1	chr3	33482078	-	116	222	2987	1067	0.522522523	11106	17481
ATP5J	chr21	27107343	-	95	182	3743	948	0.521978022	8879	12497
CTNND1	chr11	57529290	+	75	146	2355	137	0.51369863	1071.5	966.5
WNK3	chrX	54384437	-	78	153	1326	325	0.509803922	4237	353
GPX1	chr3	49396032	-	127	251	1509	443	0.505976096	12864	28961
FTH1	chr11	61735131	-	128	253	2741	939	0.505928854	104816.5	352473.5
LRRC16A	chr6	25279656	+	137	271	2425	968	0.505535055	8057.5	677.5
HAUS4	chr14	23426230	-	119	237	900	200	0.502109705	3852	2205.5
CCNG1	chr5	162864575	+	83	168	1774	473	0.494047619	67855	37863.5
IPP	chr1	46216321	-	129	264	628	278	0.488636364	803	2266
CTNNA1	chr5	138089350	+	106	218	1850	712	0.486238532	32943.5	45594
EDA2R	chrX	65859107	-	81	167	475	261	0.48502994	1666	4100
GPC3	chrX	133119921	-	402	836	893	93	0.480861244	11869	1661
DDB2	chr11	47236493	+	129	270	1599	299	0.477777778	6276.5	4773.5
UBC	chr12	125399136	-	177	371	3446	1321	0.477088949	52653	152364.5
INO80	chr15	41408551	-	110	234	2518	1018	0.47008547	4906.5	4588.5
ASCC3	chr6	101329239	-	96	207	1572	790	0.463768116	15536.5	21634
SPRYD4	chr12	56862301	+	121	262	1187	288	0.461832061	1900.5	1063.5
BIRC8	chr19	53794874	-	100	217	8	50	0.460829493	0	0
BBS9	chr7	33169144	+	96	209	1680	601	0.459330144	6806	2724
TLR5	chr1	223316623	-	501	1090	286	163	0.459633028	231.5	50.5
HDAC1	chr1	32757741	+	125	275	1490	644	0.454545455	11193.5	13327
PLAG1	chr8	57123837	-	93	205	2789	1101	0.453658537	2939	3881
PIAS2	chr18	44497467	-	127	280	1822	768	0.453571429	6561.5	5514.5
DNMT3B	chr20	31350191	+	164	362	4348	597	0.453038674	202970.5	1269.5
TRIAP1	chr12	120884214	-	113	253	3263	1018	0.446640316	5638.5	5246
CLEC2A	chr12	10084945	-	75	170	11	12	0.441176471	13	1
PPFIA3	chr19	49631584	+	174	398	617	123	0.43718593	2425	577.5
GABRB3	chr15	27012382	-	101	235	127	28	0.429787234	56045	154.5
RPL37A	chr2	217363578	+	106	247	2920	465	0.429149798	200868	185353
PCCA	chr13	100741269	+	110	258	2298	841	0.426356589	3973.5	2291.5
FBXL3	chr13	77601329	-	84	200	1605	918	0.42	1555	5988.5
C9orf43	chr9	116172966	+	109	261	2586	618	0.417624521	56.5	235
DYNC2H1	chr11	102980160	+	271	650	1236	423	0.416923077	1766	4996
FMN1	chr15	33486896	-	294	708	493	215	0.415254237	855	181.5
RPS19	chr19	42363988	+	111	274	2158	794	0.405109489	76830	84415.5
GLT6D1	chr9	138531385	-	117	289	12	32	0.404844291	9	0
PINX1	chr8	10697385	-	108	272	1594	475	0.397058824	1882.5	547.5
KLRG1	chr12	9102640	+	105	266	2789	720	0.394736842	499.5	60
HARS	chr5	140071121	-	90	229	1856	656	0.3930131	4753.5	10082
SNRPN	chr15	25200152	+	108	275	2925	968	0.392727273	37156.5	9293
EFHC2	chrX	44202922	-	63	167	158	85	0.377245509	558.5	83.5
PSMF1	chr20	1099241	+	132	352	1594	862	0.375	7217	15785.5
CHERP	chr19	16653340	-	87	233	2612	887	0.373390558	12401.5	7346.5
CHD2	chr15	93443565	+	48	131	1952	144	0.366412214	9262.5	15666.5
C1orf88	chr1	111889242	+	164	444	707	200	0.369369369	NA	NA
RFC4	chr3	186524289	-	104	283	1792	578	0.367491166	13254.5	4660
LTN1	chr21	30365276	-	72	202	1645	565	0.356435644	6388.5	6610
VTA1	chr6	142468394	+	73	205	1179	296	0.356097561	8133.5	8941
FUT9	chr6	96463860	+	440	1233	1512	178	0.356853204	3215.5	0
PLN	chr6	118869461	+	21	61	4	12	0.344262295	0	1183.5
LDB1	chr10	103874702	-	98	280	1271	647	0.35	12518	18044.5
CLEC3A	chr16	78056412	+	122	358	28	47	0.340782123	4	65.5
ACTR3B	chr7	152456873	+	92	274	1691	616	0.335766423	2626.5	461
CGA	chr6	87804823	-	75	230	7	25	0.326086957	1.5	4
FAM156A	chrX	53024643	-	56	175	823	185	0.32	13	31
COBL	chr7	51384495	-	518	1620	2385	175	0.319753086	5870.5	14.5
C2orf51	chr2	88824169	+	154	493	12	63	0.312373225	NA	NA
OR6B3	chr2	240985488	-	211	677	18	87	0.311669129	1	0
THNSL2	chr2	88469917	+	277	895	484	214	0.309497207	597.5	999
TRPM1	chr15	31362235	-	129	429	10	57	0.300699301	38	0
KRCC1	chr2	88355247	-	69	233	1521	537	0.296137339	1356	3771
C14orf101	chr14	57046544	+	93	338	1752	654	0.275147929	NA	NA
EDDM3A	chr14	21214051	+	131	493	10	30	0.265720081	1	0
MED4	chr13	48669203	-	67	260	1387	589	0.257692308	3916.5	4646
GNG4	chr1	235814053	-	272	1156	1523	148	0.235294118	8275.5	16.5
LECT2	chr5	135290722	-	68	298	6	28	0.228187919	8.5	2.5
NHLH2	chr1	116383746	-	301	1323	445	77	0.227513228	337	3.5
IQGAP2	chr5	75699149	+	314	1393	1313	238	0.225412778	48558	639
CDH1	chr16	68771255	+	179	848	2021	207	0.211084906	81065.5	2
OR52K2	chr11	4470525	+	91	457	7	45	0.199124726	1.5	0
TIFAB	chr5	134788088	-	197	1027	8	61	0.191820837	0	0
PTPRN	chr2	220174369	-	296	1554	478	71	0.19047619	3005.5	81.5
SLC26A5	chr7	103061968	-	79	420	9	15	0.188095238	35	3
KRTAP10-2	chr21	45971387	-	222	1180	50	115	0.188135593	5	0
BCL2A1	chr15	80263510	-	61	367	14	22	0.166212534	1	0.5
FAM65B	chr6	24877540	-	97	609	75	32	0.159277504	6754	161
CLRN3	chr10	129691210	-	52	343	6	22	0.151603499	0	6
FAM101A	chr12	124796254	+	121	796	14	62	0.15201005	17.5	7.5

KRTAP10-7	chr21	46020497	+	139	934	30	99	0.14882227	1	0
IGFL3	chr19	46627930	-	123	832	11	24	0.147836538	14	20.5
C8orf22	chr8	49984900	+	38	276	11	31	0.137681159	4	0
GAS7	chr17	9963131	-	79	606	15	63	0.130363036	2296	12999
LALBA	chr12	48963848	-	100	951	9	31	0.105152471	0.5	0
KIAA1671	chr22	25465936	+	80	871	11	54	0.09184845	5985	4747.5
CADM2	chr3	85008132	+	107	1178	2730	335	0.090831919	2119	3
RUNX1T1	chr8	93107670	-	116	1473	719	46	0.078750849	8011	4819.5

SUPPLEMENTARY FIGURE LEGENDS AND TABLE LEGENDS

Figure S1. Validation of H9 hESCs and H9 hMSCs using pluripotent and differentiation markers respectively.

(A) Immunofluorescence analyses of H9 hESCs for pluripotent markers, OCT4, NANOG, SOX2, SSEA-4, and TRA-1-80 are shown. Expression of pluripotent cell surface markers, SSEA-4 and TRA-1-80 were also examined by flow cytometry analysis. The percentage of SSEA-4 positive and TRA-1-80 positive cells are shown.

(B) Differentiation status of H9 hMSCs was validated by flow cytometry analysis for MSC surface markers, CD29, CD44, CD73 and CD105. The percentage of positive cells were shown.

Figure S2. p21 expression in N1-hiPSCs is very low even upon p53 activation by DNA damage.

N1-hiPSCs and N1-hiPS MSCs were treated with the indicated concentration of etoposide (A) or hydroxyurea (B) for 24 hrs and harvested for Western blotting. 50 μg of protein lysate was loaded in each lane. The passage number of N1-hiPSCs is P37 (A) and P36 (B), and the passage number of N1-hiPS MSCs is P9 (A) and P8 (B).

Figure S3. Post-translational modifications of p53 in hESCs and hMSCs.

(A) Detection of p53 mono-methylation at K372. Cell lysates were immunoprecipitated with antibody specific for p53 mono-methylated at K372. Immunoprecipitates were separated by SDS-PAGE and detected with p53 antibody by Western blotting analysis. The levels of mono-methylation at K372 are similar between H9 hESCs and hMSCs. H1299 (*p53*-null, human non-small cell lung carcinoma cell line) was used as a negative control. The passage numbers of H9 hESCs and hMSCS are P40 and P11 respectively.

(B) Detection of p53 acetylation at K120 and K382. Cells were treated with 0.5 μM Trichostatin A (TSA) and 5 mM Nicotinamide for 16 hours, and protein lysates were harvested for Western blotting analysis. The levels of acetylation at K120 and K382 are similar between H9 hESCs and hMSCs. The passage numbers of H9 hESCs and hMSCs are P28 and P9 respectively.

Figure S4. Translation analysis of *p21* mRNA in WI-38 cells

(A) Translation analysis of *p21* mRNA in WI-38 normal human fibroblasts. Representative polysome profile from WI-38 cells indicating monosome and polysome fractions is shown in the left panel. qRT-PCR analysis shows the percentage of *TBP* and *p21* mRNA in each fraction in the right panels.

(B) The expression of *p21* mRNA and proteins is similar between H9 hMSC and WI-38 cells. Cell lysates from H9 hMSC and WI-38 cells were analyzed by qRT-PCR (left) and Western blotting (right). The passage number of H9 hMSCs is P6 (PCR) and P8 (WB).

Figure S5. p53 localizes in the nucleus in mouse E14 mESCs and human H9 hESCs

(A) Immunofluorescence assay of mouse E14 mESCs was performed with the indicated antibodies. A merged image and Hoechst staining are also shown. OCT4 is an ES cell marker that localizes in the nucleus (scale bar: 20 μm).

(B) H9 hESCs were infected with lentivirus carrying inducible pTRIPZ-myc-p53. Cells were selected with 1 μg/ml puromycin, and p53 was induced by treating cells with 1 μg/ml DOX for 3 days. Immunofluorescence assay was performed with the indicated antibodies. A merged image and Hoechst staining are also shown. Note that myc-p53 is surrounded by the nuclear envelope marker, lamin B1 (LMNB1) (scale bar: 20 μm).

Figure S6. DZNep triggers degradation of EZH2 and SUZ12.

H9 hESCs and hMECs were treated with DZNep at the indicated dose for 2 days. Proteins were harvested for Western blotting analysis with the indicated antibodies. The passage numbers of H9 hESCs and hMSCs are P35 and P9 respectively.

Table S1. Coordinates of p53 ChIP-Seq peaks in H1 hESCs.

Putative p53 candidate genes in H1 hESCs are listed based on p53 ChIP-Seq data set.

Table S2. The comparison of H3K27me3 marks, H3K4me3 marks and mRNA levels of p53 candidate target genes between H1 hESCs and H1 hMSCs.

p53 target gene candidates are ranked by the levels of H3K27me3 marks in H1 hESCs compared to H1 hMSCs [H3K27me3 ratio (ESC/MSC)]. Top 10 p53 target gene candidates (excluding p21) are shown in red.

SUPPLEMENTARY MATERIALS AND METHODS

Primers used for Quantitative Real-Time PCR

Human primers

Gene	Forward primer	Reverse primer
TP53	GAGGTTGGCTCTGACTGTACC	TCCGTCCCAGTAGATTACCAC
CDKN1A (p21)	TCTTGTACCCTTGTGCCTCG	AGAAGATCAGCCGGCGTTTG
OCT4	TCAGCCAAACGACCATCTGCC	TTCTCTTTCGGGCCTGCACG
NANOG	AATGGTGTGACGCAGAAGGCC	TTGGAAGGTTCCCAGTCGGG
GDF3	TTTCCCAAGCTTCCTCCTGCC	CGGGGGTTGTCATTCCAATCC
LIN28	ACCAGCAGTTTGCAGGTGGCT	TCATGGACAGGAAGCCGAACC
GATA4	CGCCCCCCGGTGCCTACGAG	CGCCTCCGGACGCAGAGCCC
GATA6	CGCCTGCGGGCTCTACAGCAAG	GTTCACCCTCGGCGTTTCTGCG
CDX2	TGGCTCACGGCCTCAACGGTGG	GGTTGAGCGTTTGCAGCAGCCC
SGMS2	TCCTACGAACACTTATGCAAGAC	CCGGGTACTTTTTGGTGCCT
PAPPA	AGAGGTCTCCGGCAGTGAT	AGCGTGGGTCTTTGTTGTCT
STK32B	GGGACTGTGAAACTCTACATCTG	ACCGTCGCTATGTTGAAGTCT
PTPRM	ATGAGGGGACTTGGGACTTG	CATGTGCTATACGGCTCATCA
TRANK1	GCTGGATCGACAGTCTCGG	TGCTGAATCCTGATAGGTCCTTA
GAS6	CTCGTGCAGCCTATAAACCCT	TCCTCGTGTTCACTTTCACCG
MEIS2	AAGAGGCATTTTCCCCAAAG	CCTGTGTCTTGCGCTAACTG
IER5	AGGCTCATCGCATCGTCAG	CGCTCAGGTAGACTTGGCG
PHLDA3	GACCCTCGTGTCCTAAACCA	CTTACCACATGGAGCACAGC
SUSD1	TATGGATTTGTAGGGAACGGGA	ATCGTTGGGAATGAATGTCTTGT
ACTA1	GGATTCCTATGTGGGCGACGA	ACATGGCTGGGGTGTTGAAGG
TBP	CGCCGAATATAATCCCAAGC	TCCTGTGCACACCATTTTCC

Mouse primers

Gene	Forward primer	Reverse primer
Tp53	CCCCTGTCATCTTTTGTCCCTT	GGGAGGAGAGTACGTGCACATAA
Mdm2	ATTGCCTGGATCAGGATTCAGTT	ACCTCATCATCCTCATCTGAGA
CyclinG1	GGCTTTGACACGGAGACATT	TCGCTTTCACAGCCAAATAA
Lif	CCCCATTTGAGCATGAACTT	AGCAGCAGTAAGGGCACAAT
Puma	ACCTCAACGCGCAGTACG	CACCTAGTTGGGCTCCATTT
Noxa	GGAGTGCACCGGACATAACT	TTGAGCACACTCGTCCTTCA
Bax	TGAAGACAGGGGCCTTTTTG	AATTCGCCGGAGACACTCG
p53Dinp1	AAGTGGTCCCAGAATGGAAGC	GGCGAAAACTCTTGGGTTGT
Pidd	GACACACAACCGCCTAGAAAG	CAGGTTTCCAATCTCAGAGGGTA
Wdr1	GGGCGATCATTTCCTCTACA	CAATGTAGAATCCGCTGGGT
Tigar	CGCTTCGCCTTGACCGTTAT	ACCCAGTCTCCGAAAGGGG
Aldh4a1	TGACCAATGAGCCCATCTTAGC	AGGGCGACAACTGGTACTGT
Slc2a9	ATCATTGTCCCTGCCTTGGTC	TGACCTGCCAGCGGACAAA
Sesn1	CGGACCAAGCAGGTTCATCC	TGATGTTATCCAGACGACCCAAA
Sesn2	CCGAAGGATCTCTTCCACTG	AGTGCCATTCCGAGATCAAG
Gamt	AAGCCAGTGATGGAGCGTT	ATACCGAAGCCCACTTCCA
Lpin1	CATGCTTCGGAAAGTCCTTCA	GGTTATTCTTTGGCGTCAACCT
Msh2	CCGCGAGGTGTTCAAGACC	ACTTCAACTCGATACTGGCGAA
Pcna	TCAGGTACCTCAGAGCAAACG	AAGTGGAGAGCTTGGCAATG
Gapdh	AGGTCGGTGTGAACGGATTTG	TGTAGACCATGTAGTTGAGGTCA

The sequences used for RNA Interference

si-p53(1)	5’- GUAAUCUACUGGGACGGAA dTdT -3’
si-p53(2)	5’- GGUGAACCUUAGUACCUAA dTdT -3’

Negative control siRNA was purchased from Dharmacon (ON-TARGET plus Non-targeting pool).

The antibodies and primers used for Chromatin Immunoprecipitation (ChIP) Analysis

Antibody	Company
p53	Santa Cruz (DO-1)
Histone H3K27me3	Millipore #07-449

Histone H3K4me3	Abcam ab12209
Pol II	Santa Cruz (H-224)
SUZ12	Cell Signaling #3737
mouse IgG	Santa Cruz

Amplicon Name	Forward primer	Reverse primer
-2965	CCGGCCAGTATATATTTTTAATTGAGA	AGTGGTTAGTAATTTTCAGTTTGCTCAT
-2283	AGCAGGCTGTGGCTCTGATT	CAAAATAGCCACCAGCCTCTTCT
-1391	CTGTCCTCCCCGAGGTCA	ACATCTCAGGCTGCTCAGAGTCT
-969	GGCAAAAGTCCTGTGTTCCAA	GAGAAGTCTGGTGACCTAACTCAGATC
-402	CCTGATCTTTTCAGCTGCATTG	GCCCCCTTTCTGGCTCA
-20	TATATCAGGGCCGCGCTG	GGCTCCACAAGGAACTGACTTC
+182	CGTGTTCGCGGGTGTGT	CATTCACCTGCCGCAGAAA
+507	CCAGGAAGGGCGAGGAAA	GGGACCGATCCTAGACGAACTT
+4001	AGTCACTCAGCCCTGGAGTCAA	GGAGAGTGAGTTTGCCCATGA
+4991	CCAGGGCTGCGATTAGGAA	GTGTCCCTCATGGGTGTGAAT
+5794	CTGGAGACTCTCAGGGTCGAA	CACATGTCCGCACCTGTCAT
+7011	CCTGGCTGACTTCTGCTGTCT	CGGCGTTTGGAGTGGTAGA
+7878	CCAGCTGGGCTCTGCAATT	GCTGAGAGGGTACTGAAGGGAAA
